# The relationship between financial disruption during the COVID-19 pandemic and mental health: A systematic review and meta-analysis

**DOI:** 10.1177/22799036251395263

**Published:** 2025-12-23

**Authors:** Thomas Richardson, Samantha Ashworth, Monica Sood, Eva McKell, Nick Maguire, Nisreen A. Alwan, Dianna Smith

**Affiliations:** 1 School of Psychology, University of Southampton, UK; 2 School of Primary Care, Population Sciences and Medical Education, Faculty of Medicine, University of Southampton, Southampton, UK; 3 University Hospital Southampton NHS Foundation Trust, Southampton, UK; 4 NIHR Applied Research Collaboration Wessex, Southampton, UK; 5 School of Geography and Environmental Science, University of Southampton, UK

**Keywords:** COVID-19, pandemic, mental health, financial difficulties

## Abstract

**Objective:**

Financial difficulties are associated with poor mental health. This paper aimed to systematically review the impact of COVID-19 related financial difficulties on mental health in adults.

**Methods:**

A systematic search was conducted across Web of Science, Medline, and PsycINFO, from March 2020 to March 2023 to identify studies examining the mental health impact of COVID-19 related financial disruption in adults. We performed two meta-analyses to quantify the effect of income loss due to the pandemic on anxiety and depression. Studies were rated using the Quality Assessment Tool for Observational Cohort and Cross-Sectional Studies from the National Heart, Lung and Blood Institute was used.

**Results:**

A total of 2659 papers were identified of which 76 (59 cross-sectional and 17 longitudinal) met inclusion criteria. The results show that COVID-19 related financial disruption (income loss and financial stress) negatively impact mental health across a range of adult populations globally, including the general population, students, and other specific groups. The meta-analyses examined data from 278,854 participants from 15 studies indicated that those who lost income reported greater anxiety levels than those who did not experience income loss. Similarly for 268,128 participants across 16 studies, a meta-analysis showed greater depression symptoms for those experiencing income loss.

**Conclusion:**

COVID-related financial constraints, both objective and subjective, are associated with poor mental health outcomes (particularly anxiety and depression) in various populations around the world. The results highlight the need for targeted clinical interventions for those experiencing mental health problems linked to financial problems during global crises.

## Significance for public health

Previous research has demonstrated that financial difficulties lead to poor mental health at a population level. This systematic review and meta-analysis demonstrate that the covid-19 pandemic impacted mental health in a range of populations globally, due to the financial disruption it caused. Income loss during COVID-19 was associated with more severe symptoms of anxiety and depression. Both objective and subjective financial strain are associated with poor mental health. Whereas most of the research was cross-sectional, some studies demonstrated a longitudinal impact of covid-109 related financial disruption. Targeted interventions may help those with finance-related mental health problems.

## Introduction

The World Health Organisation (WHO) declared that the coronavirus (COVID-19) outbreak reached global pandemic status on 11 March 2020. Over 3 years later, on 5 March 2023, the WHO announced that COVID-19 no longer constituted a public health emergency of international concern (PHEIC). This pandemic drastically altered people’s lives and has had profound consequences on society in terms of physical health, mental health, and the economy. From research regarding previous pandemics, such as that of the severe acute respiratory syndrome (SARS, 2002–2003), it is understood that the diverse and far-reaching effects of pandemics are likely to endure beyond the period of the pandemic.^1^ The effects of the COVID-19 pandemic on mental health have been suggested to follow three main routes, namely: the disease itself, the associated imposed quarantine and social measures, and the economic consequences of the pandemic.

An established and expanding body of research has focussed on the relationship between mental health and economic concepts such as socioeconomic status (SES) and unemployment. While research has focussed broadly on SES and mental health,^2,3^ recent research has focussed on specific socioeconomic variables. For example, studies show that financial hardship (difficulty meeting financial obligations) is a stronger predictor of depression than other socioeconomic variables such as educational attainment and household income while controlling for differences in household demographic composition, size, and subsequent financial requirements.^4^ Research has also distinguished objective and subjective financial impact, with the former describing measurable financial impact (e.g. income loss, debt amount) and the latter describing perceived financial impact (e.g. financial stress/worry). Research shows that subjective financial worries have a greater impact on mental health than objective economic impact.^5^

A recent systematic review assessed the impact of the COVID-19 pandemic, previous pandemics, previous epidemics, and the 2008 economic crisis on mental health.^6^ The review showed that socioeconomic factors and unemployment resulting from the 2008 economic crisis had negative effects on mental health, including an increase in affective disorders. The main risk factors mediating the effects of the economic crisis on poor mental health included unemployment, indebtedness, precarious working conditions, inequalities, housing instability and lack of social connectedness.^6^ Another review examining the impact of economic decline on mental health found that while the effects of economic crises most negatively impacted individuals who were considered poor, less educated, or unemployed, these also affected the general population and individuals in employment, indicating that the negative impact on mental health is experienced widely by diverse groups.^7^

### Current review

While several systematic reviews have examined the psychological impact of the COVID-19 pandemic,^8–11^ to our knowledge, no systematic review and meta-analysis has investigated the relationship between COVID-19 related financial changes and mental health. A recent review looks at the association between socioeconomic condition indicators (e.g. education, economic factors) and anxiety and depression^12^; the present review offers a more focussed and detailed examination of how financial change during the pandemic relates to mental health. As COVID-19 has caused significant detrimental economic consequences on individual, community, and wider societal levels, and given the established association between financial hardship and mental health difficulties, it is imperative that this area is examined and understood to inform local and national policy and intervention, resource, and support planning.

The objective of this review is to synthesise the existing evidence from cross-sectional and longitudinal quantitative studies that examine the relationship between COVID-19 related financial change and mental health.

## Method

### Databases and search terms

The review protocol was prospectively registered on Prospero (CRD42023400004) prior to conducting the systematic searches. We followed the Preferred Reporting Items for Systematic Reviews and Meta-Analyses (PRISMA) guidelines^13^ (see checklist in Supplemental Information). Three electronic databases, Web of Science, Medline and PsycINFO, were searched in March 2023. The following search terms were used to search all fields: (poverty OR ‘financ* difficult*’ OR ‘financ* hardship’ OR debt OR ‘financial stress’ OR income) AND (COVID* OR coronavirus OR SARS-CoV* OR ‘severe acute respiratory syndrome coronavirus*’) AND (‘mental health’ OR ‘mental illness’ OR ‘mental disorder’ OR depression OR anxiety OR stress OR distress OR ‘psychological disorder’ OR ‘psychological wellbeing’ OR ‘psychological well-being’). The following limiters were set for all searches: scholarly (peer reviewed) journals published between March 2020 and March 2023. The age limiter was set to include studies related to adults (18+ years) only. Language was restricted to the English language due to time and translation constraints.

### Inclusion and exclusion criteria

Papers were included if they: (a) were original quantitative studies published in a peer-reviewed journal (b) used a cross-sectional or longitudinal design, and (c) examined the relationship between mental health and financial changes during the COVID-19 pandemic in adults aged 18+ years. For the purposes of this review, *financial changes* were defined as any changes in individuals’ financial situations during the COVID-19 pandemic, including objective financial changes (e.g. reduced income) and subjective financial stress or worry (e.g. concern over debt repayment). Financial changes during COVID-19 must have been explicitly measured by a minimum of one question regarding financial situation (e.g. ‘over the last 2 weeks, to what extent have you experienced financial distress related to COVID-19?’). Studies which investigated job loss without specified financial changes were not included due to the scope of this review and the fact that several countries’ governments subsidised wages during the COVID-19 pandemic, such as the UK government’s Coronavirus Job Retention Scheme, also known as the Furlough Scheme. Inclusion required that mental health be considered using a standardised measure, preferably the full measure but shortened versions used in previous research with demonstrated validity and reliability were also included. We sought to conduct a comprehensive review and therefore used broad inclusion criteria of any financial change during COVID-19 and any mental health outcome (e.g. anxiety, depression, stress) including symptoms and pre-existing conditions. Reviews, meta-analyses, and commentaries/letters were excluded, as were papers that did not meet the inclusion criteria.

### Search procedure

We used the software Rayyan^14^ to conduct the screening process. We first screened titles against the inclusion and exclusion criteria, and then abstracts. Abstracts that were retained were assessed for eligibility. A record was kept of the reasons for rejection. For abstract and full paper review, the most prevalent reasons for rejection included: multiple reasons, no financial measure, no standardised mental health measure and the relationship between COVID-19 financial changes and mental health not being measured. Due to the large volume of studies identified in the initial search and time constraints, a second reviewer screened a random 10% of the studies at abstract stage (following recommendations^15^). The inter-rater reliability was calculated using Cohen’s kappa, which indicated ‘substantial’ agreement (κ = 0.830). Finally, a citation search was performed for all included papers.

### Data extraction and analysis

Relevant information from each paper was extracted (e.g. design, COVID-19 phase, population description, sample size, recruitment strategy, data collection method, measures of mental health/financial change measure, analyses). Data was extracted by one of the authors and then verified another author. We then conducted a narrative synthesis following guidance by the Cochrane Consumers and Communication Review Group (Ryan, R., 2013). Meta-analysis was conducted using Comprehensive Meta-Analysis version 4.0.

We also conducted two meta-analyses on a portion (*n* = 31) of the cross-sectional studies to examine the association between income loss (vs no income change) during the pandemic and anxiety and depression. A random effects model was utilised to calculate pooled effect sizes. Following recommendations^16^ heterogeneity was assessed using a number of statistics (Cochran’s Q, Tau,^2^ and I^2^) to provide a comprehensive account. Egger’s test was used to assess publication bias.

### Quality assessment

Following the guidelines^16^ including the PRISMA 2020 statement,^17^ we assessed the internal validity and risk of potential bias of the included studies. We used the Quality Assessment Tool for Observational Cohort and Cross-Sectional Studies from the National Heart, Lung and Blood Institute^18^ as its criteria were relevant to the studies included. This tool has not been designed to provide an overall quality score, but to elicit the key concepts for evaluating the internal validity of a study; the tool guidelines indicate that the ratings be used to consider the risk of potential for selection bias, information bias, measurement bias, or confounding to determine the ability of the study to draw conclusions about the effects of the exposures on outcomes.^18^ Twenty-five percent of the papers were reviewed by an independent rater (agreement score: 89.17%); disagreements/uncertainties were discussed between the reviewers and with a third reviewer, if necessary. All studies were included in the review regardless of their quality rating and the implications of this are considered in the discussion.

## Results

**Figure 1.**
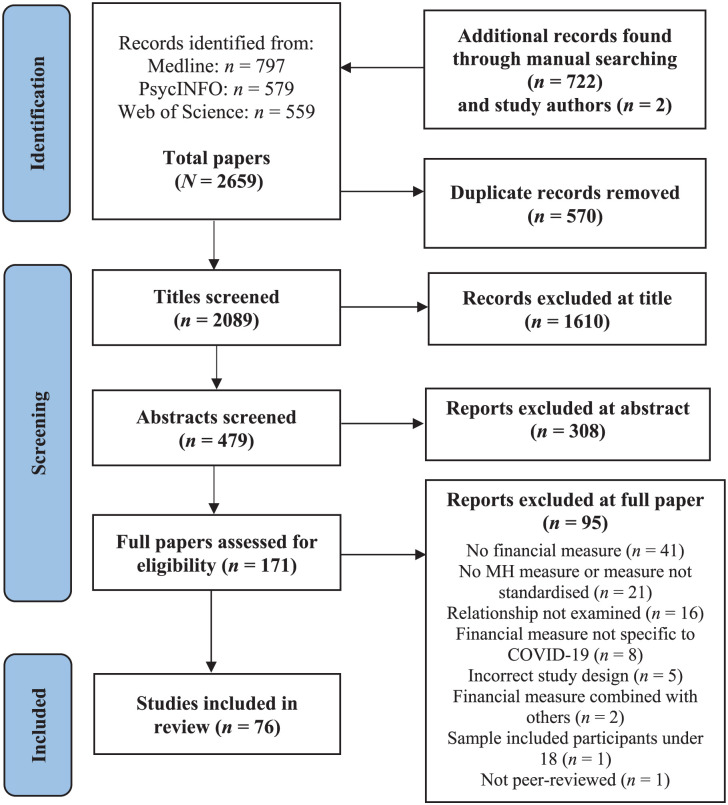
PRISMA flow diagram.

The database searches yielded 1935 papers (Figure 1). Of these, 383 abstracts were screened, and 121 full papers reviewed. A further 715 papers were retrieved by hand and citation searching; 627 of these were rejected at title and 46 at abstract-screening, leaving 42 full papers reviewed, of which six met inclusion criteria. In total, 76 studies (59 cross-sectional, and 17 longitudinal) were included for review.

### Quality assessment

Of the 59 cross-sectional studies included, 32 were rated as good, 23 as fair, and 4 poor (see Supplemental Table S1). Most (*n* = 50) did not include a power analysis or clear power description to justify their sample size, though many had large sample sizes (with an average of 8747 participants across all studies). Just over half (*n* = 33) examined different levels of the exposure variable (i.e. financial change). Most studies (*n* = 57) did not use exposures that were clearly defined, valid, and reliable, though all included outcome measures that were, and most (*n* = 54) controlled for potential confounds (e.g. sociodemographic characteristics such as age, gender, education level).

Of the 17 longitudinal studies included, 6 were rated as good, 7 as fair, and 4 poor (see Supplemental Table S2). Most (*n* = 15) did not include a power analysis or description to justify their sample size. All assessed COVID-19 related financial changes at the same time as mental health outcomes were measured, not before, precluding claims regarding directionality. Most (*n* = 13) did not assess financial changes due to COVID-19 more than once, limiting assessment of changes over time. Despite these limitations, most studies (*n* = 14) controlled for potential confounders (e.g. pre-COVID-19 income, current income, employment, and sociodemographic characteristics); all included outcome measures that were clearly defined, valid and reliable; and all but one included exposure measures that were clearly defined, valid, and reliable.

### Data extraction and study characteristics

Supplemental Tables S3–S5 summarise the data extracted from the 59 cross-sectional studies. Supplemental Table S6 summarises data extracted from the 17 longitudinal studies. The studies recruited from over 30 countries, with four recruiting from multiple countries.

The total sample size across the 59 cross-sectional studies was 513,308 (range 84–94,550). Twenty-six of these recruited general adult population samples (Supplemental Table S3), 10 recruited University students (Supplemental Table S4), and 23 recruited other, specific samples such as young/older adults, mothers, and working adults (Supplemental Table S5). All recruited between 2020 and 2021 and were published 2020–2023. Most (*n* = 53) cross-sectional studies collected data in 2020, with the majority between March and June in the early phases of the pandemic. In terms of COVID-19, this was a time of significant uncertainty, increasing cases and COVID-19-related deaths, with local and national restrictions worldwide. The remaining studies collected data primarily before and during the second COVID-19 wave; nine studies collected data in 2021, during the easing of lockdown restrictions and later waves.

The total sample size across the 17 longitudinal studies was 31,680 (range 241–6057, Supplemental Table S6). Eleven recruited general adult population samples. The remaining six recruited specific populations, including parents^19,20^, middle- and older-aged adults,^21,22^ young adults,^22^ and working adults.^23^ Most longitudinal studies were prospective, and only one was ambispective.^24^ Most (*n* = 13) longitudinal studies commenced data collection in 2020. Of these, most collected data between March and June 2020. The remaining studies which began in 2020 continued to collect data in 2021 and 2022, meaning that these studies collected data during the easing of lockdown and following the introduction of COVID-19 vaccines. Finally, four studies began earlier in the pre-pandemic phase, between 2015 and 2018, and ended between May 2020 and March 2021. These utilised various data collection periods, from 12 to 15 days between surveys and surveys administered over 5 years. Most studies were conducted over 2–6 months.

We first report the results from the cross-sectional studies, in each of the various samples recruited, followed by the longitudinal studies. The results are organised to summarise the impact of objective financial changes (measurable financial impact, e.g. income loss, debt amount), subjective financial worries (perceived financial impact, e.g. concerns about debt repayment, financial stress), and financial hardship (difficulty meeting financial obligations, e.g. paying bills) on mental health.

### Cross-sectional studies

#### Student samples (n = 10)

##### Objective (n = 10)

Ten cross-sectional studies in student samples examined the impact of objective economic hardship, such as income loss due to the pandemic, on various mental health outcomes. Most (*n* = 9) found that financial difficulties (i.e. income loss and financial struggles, stress, and insecurity) due to COVID-19 were associated with greater chances of experiencing distress, anxiety, depression, suicidal thoughts, and traumatic stress (including post-traumatic stress disorder). Two studies found contradictory results, with one^25^ finding that depression scores were variable in those who lost income, whilst another^26^ found no association between financial difficulties and stress (though financial difficulties were associated with increased depression and anxiety).

##### Subjective (n = 2)

Two cross-sectional student studies examined the subjective economic impact on student mental health.^25,27^ Both found that greater financial stress/worries were associated with worse mental health outcomes (e.g. increased depression, anxiety, suicidal thoughts, and traumatic stress).

#### General population samples (n = 26)

##### Objective (n = 20)

Twenty cross-sectional studies in general population samples examined the objective financial impact on mental health. Eighteen found that having financial situation impacted by COVID-19, such as income loss, job loss, and general financial difficulties (e.g. inability to pay bills, problems managing debt) were associated with poor mental health including poorer psychological wellbeing, poorer quality of life, and greater psychological distress, anxiety, depression, stress, loneliness, and trauma-related distress. One study additionally found that distress mediated the relationship between financial difficulties and quality of life, with greater financial difficulties leading to greater distress and, in turn, poorer quality of life.^28^ Another found a dose-response relationship between debt management issues and depression and anxiety, with greater debt management issues leading to greater mental health difficulties.^29^ One study^30^ found that the impact of income loss on depression, stress, and anxiety was exacerbated for those who also lost their jobs due to the pandemic. Another study^31^ found that those with greater levels of anxiety and depression were more likely to have lost their jobs or income and struggle to meet financial obligations during the pandemic compared to those without anxiety and depression.

Two studies found that COVID-related financial impact (including income change) was not associated with psychological distress.^32,33^

##### Subjective (n = 7)

Seven cross-sectional studies in the general population examined the subjective financial impact on mental health. Five of these found that worries and distress about finances due to COVID-19 predicted poorer mental health, including less psychological wellbeing and greater depression, anxiety, distress. One study found that these worries mediated the positive association between deprivation and mental health disorders.^34^ Another found that participants who perceived themselves as financially vulnerable due to the pandemic reported greater distress.^35^

However, one study found that worries about the impact of COVID-19 on finances were not associated with psychological distress (though there was a non-significant trend for a positive relationship between these variables^36^).

##### Financial hardship (n = 3)

Three cross-sectional studies in the general population examined the impact of financial hardship on mental health. One of these found that difficulty meeting financial obligations due to COVID-19 was associated with greater anxiety.^37^ Similarly, one study found that difficulty paying expenses was associated with anxiety and depression in a dose-response relationship: the more financial hardship participants reported, the greater risk they had of experiencing anxiety and depression compared to those without financial hardship.^38^ Another study found that those reporting anxiety and depression were more likely to struggle to meet financial obligations compared to those without anxiety and depression.^31^

##### Other samples (n = 23)

Twenty-three studies recruited other samples, including workers, clinical samples, mothers and pregnant women, young/older adults, and other specific samples.

#### Workers (n = 10)

##### Objective (n = 7)

All seven studies found that objective financial changes due to COVID-19 (such as income loss, job loss, economic burden) were associated with a range of mental health problems such as greater stress, distress, anxiety, depression, PTSD risk, and reduced life satisfaction.

##### Subjective (n = 1)

One study examining subjective economic impact in remote workers found that financial concern was associated with greater stress, but *current* financial concern/situation did not predict *current* stress, anxiety, or depression.^39^

##### Financial hardship (n = 2)

One study^40^ found that perceived financial hardship was associated with poorer wellbeing and greater depression and loneliness in performing arts professionals. Another^41^ found that financial hardship predicted twice the risk of experiencing greater anxiety.

#### Clinical (n = 2)

##### Objective (n = 1)

One study recruited participants with a history of mental illness (e.g. depression, anxiety, personality disorder) and examined the objective economic impact on mental health.^42^ The study found that low income and having income impacted by the pandemic was associated with poorer mental health during the COVID-19 pandemic, including greater anxiety and depression symptoms and reduced wellbeing.

##### Financial hardship (n = 1)

A study in Bangladesh^43^ found that among people with underlying physical/mental health conditions, financial difficulties negatively impacted mental health.

#### Mothers and pregnant women (n = 3)

##### Objective (n = 1)

Pregnant women who lost income due to COVID-19 had greater odds of experiencing depression and anxiety.^44^

##### Subjective (n = 1)

Pregnant women who were worried about their financial situation due to COVID-19 were more likely to experience clinically significant levels of depression, even when covarying demographic variables such as income level and education.^45^

##### Financial hardship (n = 1)

In mothers and children with adversity before COVID, greater financial hardship was associated with greater maternal and child mental health problems while covarying pre-COVID mental health.^46^

##### Older adults (n = 3)

All studies reported the objective financial impact on mental health; older adults who experienced financial difficulties (including income loss and inability to make a household payment on time) reported greater emotional distress,^47^ depression, and anxiety.^48^ Older adults who expected further income losses and expected being unable to make the next house payment reported greater distress.^47^ Conversely, another study^49^ found that those who had job security, less financial change, and were able to make ends meet had better mental health, even when controlling demographic characteristics.

##### Young adults (n = 2)

Both studies examined the objective financial impact on mental health and found that young adults aged 18–35 years who lost income or job due to COVID-19 had lower psychological wellbeing^50^ and greater anxiety and PTSD (though not depression^51^).

##### Specific samples (n = 3)

Three studies recruited samples including middle income households,^52^ impoverished urban dwellers,^53^ and Black cisgender sexual minority men and transgender women.^54^ All studies examined objective economic impact on mental health, and one examined both objective and subjective impact (i.e. income loss and worries about job loss^54^). A study in Bangladesh^52^ found that middle income participants who experienced income loss or debt had greater depression and anxiety symptoms, though the impact of COVID-19-related income loss on anxiety and depression was small. Another study in Bangladesh^53^ found that income loss did not impact PTSD and depression, though having a lower household income was associated with greater PTSD severity. Finally one study^54^ found that income loss and worries about job loss were positively associated with loneliness, though not anxiety and depression.

#### Longitudinal studies

Most (*n* = 11) longitudinal studies were conducted in the general population, with two of these including samples nationally representative of residents. The remaining were conducted in older adults (*n* = 2), young adults (*n* = 1), adult workers (*n* = 1), parents of school children (*n* = 1) and mothers (*n* = 1).

##### Objective (n = 9)

Nine longitudinal studies examined the relationship between perceived objective economic impact due to COVID-19 and metal health outcomes.^19,20,24,55–59^ Of these, seven found positive associations between economic impact and depressive symptoms, and five found positive associations between economic impact and anxiety. One study^57^ found that, in both study cohorts, economic impact was more strongly associated with depression than anxiety. However, the increase in anxiety symptoms was steeper than that of depression.^57^ Only one study found that economic impact was not associated with depressive or anxiety symptoms^56^; however, it received an overall quality assessment rating of ‘poor’ due to the risk of bias, high attrition rate (54.95%), and sample variability. Regarding the other mental health outcomes studied, one study showed that COVID-19 related economic impact was associated with increased psychological distress^20^; another found that lower economic impact was associated with greater positive affect at two time points.^55^

##### Subjective (n = 7)

Seven longitudinal studies examined the relationship between subjective financial worry due to COVID-19 and mental health.^1,57,60–63^ All found positive associations between COVID-19-related financial stress and worse mental health, including depression, anxiety, and global mental health. Hertz-Palmor et al.^57^ demonstrated a positive association between financial worries and depression. This association was unique to financial worries as health-related worries were associated with general symptom load but not depression. Furthermore, this finding remained while controlling for pre-COVID-19 income which suggests that variability in depressive symptoms is only partially explained by objective financial situation and that financial stress may be a more significant predictor of depression.^57^ In terms of general mental health, Simonse et al.^1^ also conducted a mediation analysis where mental health was the dependent variable, financial stress was the mediator, and income, savings, and debts were the independent variables. This analysis found that financial stress mediated the relationship between savings and debts on the one hand, and changes in mental health on the other.

##### Financial hardship (n = 4)

All studies reported positive associations between financial hardship and poor mental health, including increased depression,^22^ negative affect,^21^ psychological distress,^23^ and maternal anxiety and depression.^19^

### Meta-analyses

We performed two random effects meta-analyses using Comprehensive Meta-Analysis version 4.0 to examine the impact of income loss due to COVID-19 on anxiety and depression.

#### Anxiety

**Figure 2.**
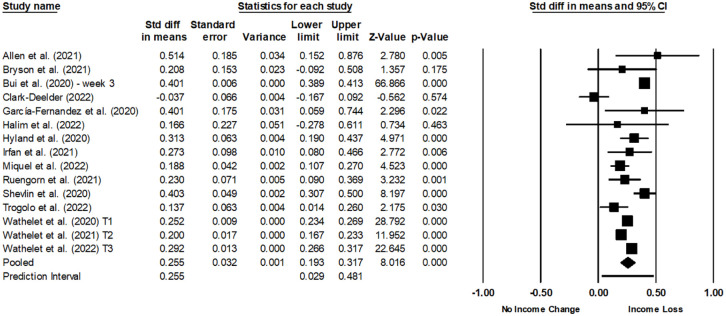
Meta-analysis statistics and forest plot for anxiety.

 Fifteen cross-sectional studies with a total of 278,854 participants were included to examine mean differences in anxiety between those who lost income and those who did not. The pooled standardised mean difference was 0.26 (95% CI: 0.19–0.32, *p* < 0.001), indicating that those who lost income had greater anxiety than those who did not (Figure 2). There was substantial heterogeneity among the included studies (*Q* = 349.36, *df*[*Q*] = 14, *p* < 0.001; *T* = 0.10, *T*² = 0.01; *I*² = 95.99%), however due to a relatively low number of studies a subgroup analysis could not be conducted to determine the cause of this heterogeneity. Egger’s test indicated no evidence of publication bias (*B*0 = −2.32, *p* = 0.16), suggesting that the asymmetry observed in the funnel plot (Supplemental Figure S1) is due to chance rather than selective reporting.

#### Depression

**Figure 3.**
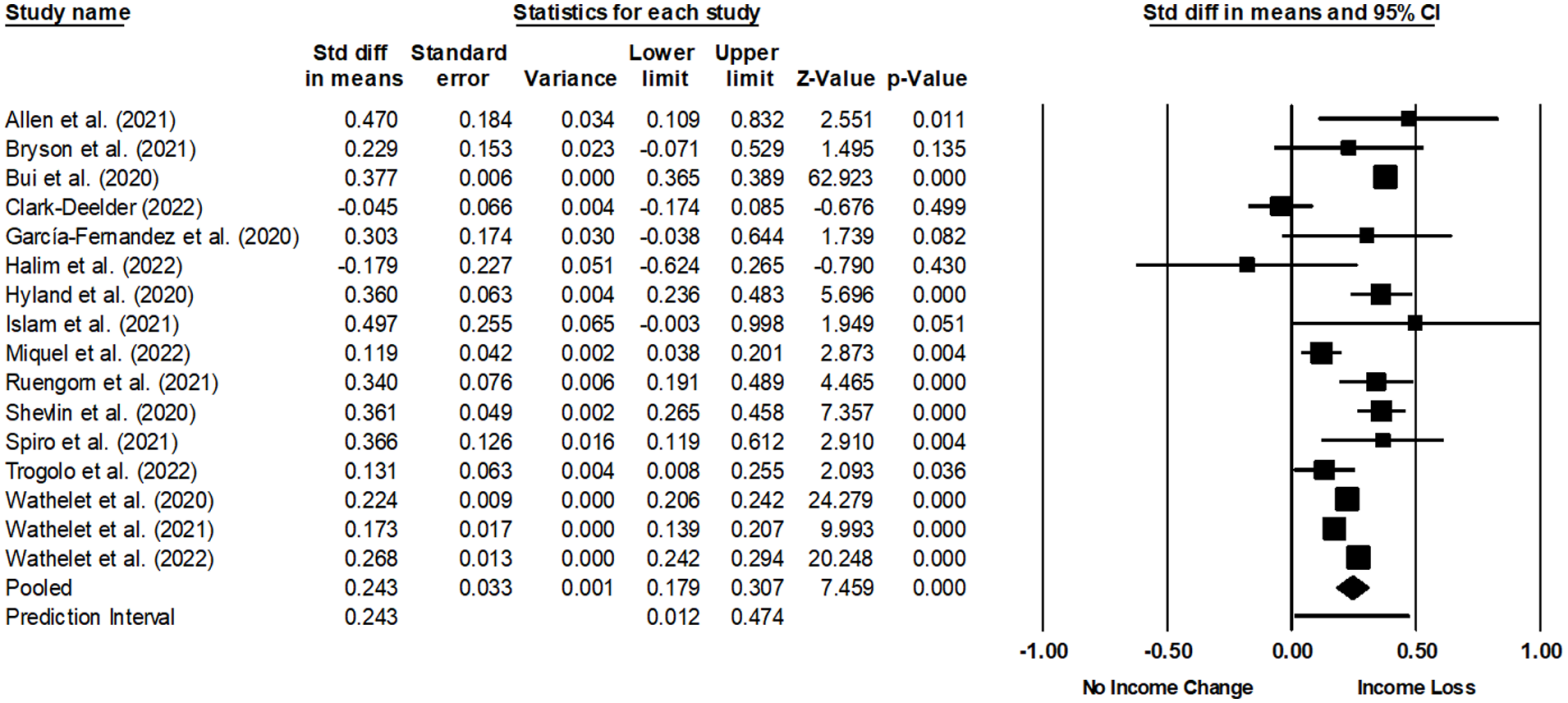
Meta-analysis statistics and forest plot for depression.

Sixteen cross-sectional studies with a total of 268,128 participants were included to examine mean differences in depression between those who lost income and those who did not. The pooled standardised mean difference was 0.24 (95% CI: 0.18–0.31, *p* < 0.001), indicating that those who lost income had greater depression than those who did not (Figure 3). There was substantial heterogeneity among the studies (*Q* = 351.95, *df*[*Q*] = 15, *p* < 0.001; *T* = 0.10, *T*² = 0.01; *I*² = 95.74%), however due to a relatively low number of studies a subgroup analysis could not be conducted to determine the cause of this heterogeneity. Egger’s test indicated no evidence of publication bias (*B*0 = −1.98, *p* = 0.20; see Supplemental Figure S2).

## Discussion

This review sought to explore the relationship between COVID-19-related financial changes and mental health. A total of 76 studies (17 longitudinal and 59 cross-sectional) met the inclusion criteria; these recruited diverse groups across the globe and examined various mental health outcomes and COVID-19-related financial disruption. The most common mental health outcomes examined were depression and anxiety. Most studies examined objective economic impact, followed by subjective financial stress and financial hardship. Together, the review findings suggest that COVID-19 related financial disruption (including income loss, financial stress, and financial difficulty) negatively impacts mental health (e.g. increased depression, anxiety, and stress) across a range of populations. The findings from the meta-analyses show that those who lost income during COVID-19 (compared to those who did not) experienced greater anxiety and depression levels.

The studies demonstrated mixed findings but evidenced an overall impact of COVID-19 on people’s mental health, independent of objective economic impact. Similar results are reported by a systematic review examining the psychological impact of COVID-19 on the general population and healthcare workers.^64^ Research on the psychological impact of COVID-19 shows that poor mental health was exacerbated in with pre-existing mental health conditions.^65^ The current review found comparable results, supporting external validity.

Forty-seven cross-sectional studies examined objective economic impact on mental health in the general population, students, and other samples including clinical groups, workers, mothers and pregnant women, older and younger adults, middle-income households, impoverished urban dwellers, and sexual minorities. Most studies (*n* = 45) found that objective economic impact due to COVID-19 (e.g. income loss, job loss, economic burden) was associated with poor mental health including greater anxiety, depression, and stress, and reduced wellbeing and quality of life. Most studies (*n* = 26) recruited general population samples across 14 countries (with one study recruiting from 59 countries), suggesting the results are generalisable to the wider population across the globe. Only three studies found somewhat contradicting results, with objective economic impact not associated with mental health in the general population^32,33^ and Black cisgender sexual minority men and transgender women.^54^ The results from Clarke-Deelder et al.’s^32^ study are particularly surprising given this study was rated as ‘good’ in quality due to the large sample, measuring valid outcomes, including a continuous measure of COVID-19-related financial impact, and covarying confounds. Cultural differences and differences in the severity of COVID-19 and country responses to the pandemic may partly explain the findings. Rahman et al.’s^33^ heterogenous sample (including patients, healthcare workers, and the public) may explain the surprising results; isolating these samples may lead to different findings (e.g. the authors found that, relative to their counterparts, those with pre-existing mental health difficulties were more likely to develop moderate to high levels of distress). Similarly, Timmins et al.’s^54^ very specific sample may exhibit different effects than those typically observed in general population samples; further work is required to closely examine these effects and the potential mechanisms involved (e.g. resilience in minoritised communities). Together, the results strongly suggest that objective economic factors such as income loss are associated with poor mental health.

Consistent with this, 10 longitudinal studies suggest that objective economic impact due to COVID-19 was associated with worsening anxiety and depression in the general population. Seven of these 10 studies received an overall quality assessment rating of ‘fair’ or ‘good’ and controlled for potential confounds (e.g. pre-COVID-19 and current income, employment, pre-pandemic mental health outcomes, and sociodemographic characteristics). Controlling for confounds enhances the internal validity by limiting the influence of variables that may affect the relationship between COVID-19-related financial changes and mental health. Most of these studies recruited general population samples, supporting the generalisability of the findings to a wider population. However, one of the seven papers which examined the relationship between objective economic impact and mental health (anxiety and depression) found no association (Hagen et al., 2023). This may be because this study had a significant risk of bias (and an overall quality rating of ‘poor’) due to several methodological limitations, including the high attrition rate (54.95%), sample variability, and discontinuous measurement of economic impact.

Nineteen studies (12 cross-sectional and seven longitudinal) suggest that subjective financial stress due to COVID-19 was associated with worsening mental health. Twelve were in the general population, supporting the generalisability of the findings. One cross-sectional study in sexual minority groups found that greater worries about job loss were associated with greater loneliness, but not anxiety and depression^54^; this study found mixed results in general, possibly due to sample characteristics. Another cross-sectional study found that financial concern was positively associated with stress, though *current* financial concern did not predict *current* mental health^39^; this suggests that the association between financial stress and mental health differs at trait (general) and state (situational) levels.

All but one of the seven relevant longitudinal studies examining subjective economic impact on mental health received an overall quality assessment rating of ‘fair’ or ‘good’, and five controlled for potential confounding variables (e.g. job and health stressors, pre-COVID-19 income, and sociodemographic characteristics). There was some evidence that COVID-19-related financial stress may be a more significant predictor of mental health than objective financial hardship. This supports Frankham et al.^5^ who found that subjective financial hardship, but not objective financial hardship, predicted mental health and Marjanovic et al.^66^ who found that the financial threat scale mediated the relationship between economic hardship and mental health as measured by the General Health Questionnaire. The evidence of subjective financial hardship being a more significant predictor than objective financial hardship is limited in this review and further research is required. Research measuring both subjective and objective financial hardship and mental health over time would be a useful addition to the literature.

Some studies (seven cross-sectional and four longitudinal) suggest that financial hardship due to COVID-19 was associated with worsening mental health. While fewer studies examined this relationship, all had a low risk of bias, controlled for potential confounds, and many (*n* = 7) received an overall quality assessment rating of ‘good’. However, most these studies recruited from specific populations (i.e. clinical groups, working adults, middle- and older-aged adults, and mothers/pregnant women), with only three cross-sectional studies in the general population, limiting the generalisability of the results. Further research on the relationship between financial hardship and mental health in the general population is required.

### Limitations of the reviewed literature

Most studies were cross-sectional and correlational, limiting causal and directional claims. The longitudinal studies demonstrate an impact of COVID-related finances on mental health over time, but there is no way to compare outcomes pre- and post-COVID. Most longitudinal studies were conducted in the first 6 months of the COVID-19 pandemic, which poses limitations as economic impact, financial stress, and financial hardship may not occur soon after a loss of income or other financial disruption. Similarly, the time between data collection points in the longitudinal studies were brief for several studies, such as Canet-Juric (2020) which had only 12–15 days between surveys. This impacted the validity of the results and contributed to this study receiving an overall quality assessment rating of ‘poor’.

There was significant heterogeneity in how studies measured COVID-19-related financial changes. Some used operational definitions of COVID-19-related financial changes with some lacking a clear definition (e.g. Baranov et al.^20^ used job loss as a proxy for economic impact). For nearly all studies, it was unclear whether financial consequences were due to getting ill with COVID, lockdown restrictions, or both. Few studies used standardised, validated financial measures. Most studies used one question to measure financial variables (e.g. income loss) and when several items were used, they did not assess the internal consistency of the scales used. Several studies also dichotomised measurements (e.g. ‘economic impact’ and ‘no economic impact’) despite using Likert scales, resulting in information loss and reduced statistical power. Another limitation of dichotomising data is that the extent of variation in outcome between groups can be underestimated, and considerable variability may be subsumed within each group.^67^ All these factors have consequences for validity and reliability, given the uncertainty that the specific financial variable is the construct being assessed, whether this assessment is accurate, and ultimately whether it is acceptable to compare these financial constructs across different studies.

All studies used self-rated measures of mental health rather than a formal diagnosis or semi-structured assessment. Participation rates were frequently unclear or unreported, as was information describing the relevant COVID-19 context and relevant restrictions. Most studies recruited self-selecting participants and, therefore, may not be representative of the target populations due to self-selection bias and non-response bias. Relatedly, as most studies utilised online methods, likely due to COVID-19 social distancing measures, people without access to the necessary devices are likely to be underrepresented. Many studies reported that there were limitations in their generalisability due to underrepresentation of specific groups, such as people from ethnic minority backgrounds and of lower socioeconomic status. Most studies reviewed were conducted in countries with a largely individualistic culture and the evidence, therefore, may be different in countries with largely collectivistic cultures.

### Strengths and limitations of the review

To the best of our knowledge, this is the first systematic review and meta-analysis examining the relationship between COVID-related financial changes and mental health. We followed PRISMA guidelines and prospectively registered the review on Prospero to enhance transparency and replicability.

Given the nature of the studies included, from this review alone, we cannot determine if the mental health impact of COVID-related financial challenges is different to that of financial problems generally. Additionally, not all financial changes assessed may be a direct impact of COVID, as some studies simply assessed financial changes during the pandemic and other factors may be involved. However, as the COVID-19 pandemic was an unprecedented and catastrophic event impacting individuals, the society, and the economy globally, with many losing their jobs and income, it was important to examine the impact of such financial challenges on mental health. An important public health consideration for future pandemics is that there may be a considerable indirect impact on mental health due to worsened finances.

Most studies were symptom based, demonstrating a short-term increase in symptoms of depression, anxiety, and more, though we cannot assume that there was increase in the prevalence of mental health problems at a community level.

Due to resource and time constraints, studies that were not written in English were excluded. This may have caused selection bias and cultural bias, which may limit the generalisability of the findings. Additionally, only three databases were searched meaning that some papers may have been missed; to partly address this, we conducted hand and citation searches to identify further articles. While one author screened and quality assessed most papers independently, a portion of the papers were screened/quality assessed by an independent rater to reduce risk of bias (10% abstracts screened and 25% papers quality assessed by independent raters). There was substantial agreement between independent raters for both screening and quality assessment, enhancing the validity and reliability of the assessment outcome. Given the subjective nature of the quality assessment tool, we suggest that the ratings (and particularly the overall ratings) be interpreted with caution. Due to a range of samples and populations studied, and the relatively small number of studies, there was insufficient data for a sensitivity analysis to determine how robust the findings are and any variables that might have impacted the meta-analysis results, and it was also not possible to examine possible causes of heterogeneity via sub-group analyses.

### Clinical implications

This review shows that the COVID-19 pandemic has negatively impacted people’s financial circumstances and mental health. Increased vulnerability to poor mental health related to COVID-19-related financial changes may have long-term consequences for both individuals and communities. Government policies which aimed to reduce the financial impact of the pandemic may have improved the mental health at a population level; however, this was not directly assessed by any of the studies here. For any future pandemics, financial assistance may help to mitigate the impact on mental health.

The results suggest that mental health practitioners should assess personal financial circumstances and incorporate these in formulations and interventions, particularly during global economic crises. Therapeutic interventions that benefit individuals facing difficult life events should be offered to people whose mental health has been impacted by COVID-19-related financial disruption. For example, Acceptance and Commitment Therapy and Compassion-Focussed Therapy seek to reduce psychological distress by increasing psychological flexibility and the ability to receive compassion, respectively; such interventions could be tailored to those experiencing financial difficulties and linked mental health problems.

### Future research

Further research is required to address the limitations and gaps identified in the existing literature. For example, research is needed to understand the mechanisms by which COVID-19-related financial changes impact mental health. As most studies reviewed were conducted in the general population, future research should investigate the effect of COVID-related financial difficulties on mental health in clinical populations (e.g. those with clinical levels of anxiety and depression) to inform clinical interventions. Studies should also utilise standardised measures of economic impact, financial stress and financial hardship which more adequately measure these constructs and their severity. While this review was conducted in the UK, there was a lack of good quality UK-based studies that met the review criteria. This paucity needs to be addressed to understand the nuances of these relationships in the context of British culture.

## Conclusions

Overall, this review demonstrates that COVID-related financial constraints (both objective and subjective) are associated with poor mental health outcomes (particularly anxiety and depression) in the general population, students, and other specific samples such as young and older adults. Given that the COVID-19 pandemic has had significant individual, societal, and global economic effects, further research is needed to continue to understand this relationship and inform relevant policy and interventions.

## Supplemental Material

sj-docx-1-phj-10.1177_22799036251395263 – Supplemental material for The relationship between financial disruption during the COVID-19 pandemic and mental health: A systematic review and meta-analysis Supplemental material, sj-docx-1-phj-10.1177_22799036251395263 for The relationship between financial disruption during the COVID-19 pandemic and mental health: A systematic review and meta-analysis by Thomas Richardson, Samantha Ashworth, Monica Sood, Eva McKell, Nick Maguire, Nisreen A. Alwan and Dianna Smith in Journal of Public Health Research

sj-docx-2-phj-10.1177_22799036251395263 – Supplemental material for The relationship between financial disruption during the COVID-19 pandemic and mental health: A systematic review and meta-analysis Supplemental material, sj-docx-2-phj-10.1177_22799036251395263 for The relationship between financial disruption during the COVID-19 pandemic and mental health: A systematic review and meta-analysis by Thomas Richardson, Samantha Ashworth, Monica Sood, Eva McKell, Nick Maguire, Nisreen A. Alwan and Dianna Smith in Journal of Public Health Research
