# Supplementary material for: The relationship between financial disruption during the COVID-19 pandemic and mental health: A systematic review and meta-analysis
Source: J Public Health Res. 2025 Dec 23;14(4):22799036251395263. doi: 10.1177/22799036251395263 (PMC12739082; doi:10.1177/22799036251395263)
Supplement: sj-docx-2-phj-10.1177_22799036251395263 – Supplemental material for The relationship between financial disruption during the COVID-19 pandemic and mental health: A systematic review and meta-analysis [file sj-docx-2-phj-10.1177_22799036251395263.docx]

**Table S1**

*Quality Assessment Ratings for Cross-Sectional Studies*

|  | 1 | 2 | 3 | 4 | 5 | 6 | 7 | 8 | 9 | 10 | 11 | 12 | 13 | 14 | 15 |
| --- | --- | --- | --- | --- | --- | --- | --- | --- | --- | --- | --- | --- | --- | --- | --- |
| Allen et al. (2021)^68^ | Yes | Yes | Yes | Yes | No | No | No | No | No | NA | Yes | NA | NA | No | Poor |
| Aruta (2021)^28^ | Yes | Yes | CD | Yes | No | No | No | Yes | No | NA | Yes | NA | NA | No | Fair |
| Aruta et al. (2021)^69^ | Yes | Yes | CD | Yes | No | No | No | Yes | No | NA | Yes | NA | NA | No | Fair |
| Bahar Moni et al. (2021)^70^ | Yes | Yes | CD | Yes | Yes | No | No | No | No | NA | Yes | NA | NA | Yes | Fair |
| Bryson et al. (2021)^46^ | Yes | Yes | Yes | Yes | No | No | No | Yes | Yes | NA | Yes | NA | NA | Yes | Good |
| Bui et al. (2021)^47^ | Yes | Yes | No | Yes | No | No | No | No | No | NA | Yes | NA | NA | Yes | Fair |
| Chung et al. (2021)^34^ | Yes | Yes | Yes | Yes | No | No | No | Yes | No | NA | Yes | NA | NA | Yes | Good |
| Clark-Deelder (2022)^32^ | Yes | Yes | No | Yes | CD | No | No | Yes | No | NA | Yes | NA | NA | Yes | Good |
| Coulombe et al. (2020)^71^ | Yes | Yes | CD | Yes | No | No | No | Yes | No | NA | Yes | NA | NA | Yes | Good |
| Davis et al. (2021)^25^ | Yes | Yes | No | Yes | No | No | No | Yes | No | NA | Yes | NA | NA | Yes | Fair |
| Dawel et al. (2020)^72^ | Yes | Yes | No | Yes | Yes | No | No | Yes | No | NA | Yes | NA | NA | Yes | Good |
| Elaidy et al. (2023)^73^ | Yes | Yes | CD | Yes | Yes | No | No | No | No | NA | Yes | NA | NA | Yes | Fair |
| ElTohamy et al. (2022)^74^ | Yes | Yes | No | Yes | No | No | No | Yes | No | NA | Yes | NA | NA | Yes | Good |
| Ertl et al. (2022)^75^ | Yes | Yes | CD | Yes | No | No | No | Yes | No | NA | Yes | NA | NA | Yes | Good |
| Frankenthal et al. (2023)^76^ | Yes | Yes | Yes | Yes | No | No | No | No | No | NA | Yes | NA | NA | Yes | Good |
| Galanza et al. (2023)^26^ | Yes | Yes | CD | Yes | No | No | No | No | No | NA | Yes | NA | NA | No | Poor |
| García-Fernandez et al. (2020)^48^ | Yes | Yes | CD | Yes | No | No | No | No | No | NA | Yes | NA | NA | Yes | Fair |
| Guerrero and Barnes (2022)^31^ | Yes | Yes | CD | Yes | No | No | No | No | No | NA | Yes | NA | NA | Yes | Good |
| Halim et al. (2022)^52^ | Yes | Yes | CD | Yes | No | No | No | No | No | NA | Yes | NA | NA | Yes | Good |
| Haque et al. (2022)^77^ | Yes | Yes | Yes | Yes | No | No | No | No | No | NA | Yes | NA | NA | Yes | Fair |
| Hyland et al. (2020)^78^ | Yes | Yes | CD | Yes | No | No | No | No | No | NA | Yes | NA | NA | Yes | Good |
| Irfan et al. (2021)^79^ | Yes | Yes | CD | Yes | No | No | No | No | No | NA | Yes | NA | NA | Yes | Fair |
| Islam et al. (2021)^53^ | Yes | Yes | CD | Yes | Yes | No | No | No | No | NA | Yes | NA | NA | Yes | Good |
| Kim (2021)^38^ | Yes | Yes | No | Yes | No | No | No | Yes | No | NA | Yes | NA | NA | Yes | Good |
| Lee (2022)^22^ | Yes | Yes | CD | Yes | No | No | No | No | No | NA | Yes | NA | NA | Yes | Fair |
| Lewis et al. (2022)^42^ | Yes | Yes | CD | Yes | No | No | No | No | No | NA | Yes | NA | NA | Yes | Good |
| Li et al. (2023)^50^ | Yes | Yes | CD | Yes | No | No | No | Yes | No | NA | Yes | NA | NA | Yes | Good |
| Li et al. (2020)^80^ | Yes | Yes | Yes | Yes | No | No | No | Yes | No | NA | Yes | NA | NA | Yes | Good |
| Liu et al (2021)^44^ | Yes | Yes | CD | Yes | No | No | No | Yes | No | NA | Yes | NA | NA | Yes | Good |
| Lu et al. (2020)^81^ | Yes | Yes | CD | Yes | No | No | No | No | No | NA | Yes | NA | NA | Yes | Fair |
| Miquel et al. (2022)^82^ | Yes | Yes | No | Yes | No | No | No | No | No | NA | Yes | NA | NA | Yes | Fair |
| Mougharbel et al. (2021)^83^ | Yes | Yes | No | Yes | No | No | No | Yes | No | NA | Yes | NA | NA | Yes | Fair |
| Nagasu et al. (2021)^36^ | Yes | Yes | CD | Yes | No | No | No | Yes | No | NA | Yes | NA | NA | Yes | Fair |
| Nelson et al. (2020)^84^ | Yes | Yes | CD | Yes | No | No | No | Yes | No | NA | Yes | NA | NA | Yes | Good |
| Odani et al. (2022)^85^ | Yes | Yes | No | Yes | No | No | No | No | No | NA | Yes | NA | NA | Yes | Good |
| Patwary et al. (2022)^41^ | Yes | Yes | CD | Yes | No | No | No | No | No | NA | Yes | NA | NA | Yes | Poor |
| Rahman et al. (2020)^33^ | Yes | Yes | CD | Yes | Yes | No | No | No | No | NA | Yes | NA | NA | Yes | Fair |
| Ruengorn et al. (2021)^86^ | Yes | Yes | CD | Yes | Yes | No | No | Yes | No | NA | Yes | NA | NA | Yes | Good |
| Sato et al. (2023)^87^ | Yes | Yes | No | Yes | No | No | No | Yes | No | NA | Yes | NA | NA | Yes | Fair |
| Sekscinska et al. (2022)^88^ | Yes | Yes | CD | Yes | Yes | No | No | Yes | No | NA | Yes | NA | NA | Yes | Good |
| Şentürk et al. (2021)^39^ | Yes | Yes | CD | Yes | No | No | No | Yes | No | NA | Yes | NA | NA | Yes | Fair |
| Sharma et al. (2022)^51^ | Yes | Yes | CD | Yes | No | No | No | No | No | NA | Yes | NA | NA | Yes | Fair |
| Shevlin et al. (2020)^89^ | Yes | Yes | CD | Yes | Yes | No | No | Yes | No | NA | Yes | NA | NA | Yes | Good |
| Shevlin et al. (2022)^29^ | Yes | Yes | CD | Yes | No | No | No | Yes | No | NA | Yes | NA | NA | Yes | Good |
| Shuster et al. (2021)^58^ | Yes | Yes | CD | Yes | No | No | No | Yes | No | NA | Yes | NA | NA | Yes | Fair |
| Simha et al. (2020)^35^ | Yes | Yes | CD | Yes | No | No | No | Yes | No | NA | Yes | NA | NA | Yes | Good |
| Spiro et al. (2021)^40^ | Yes | Yes | CD | Yes | No | No | No | Yes | No | NA | Yes | NA | NA | Yes | Fair |
| Sujan et al. (2022)^43^ | Yes | Yes | CD | Yes | No | No | No | No | No | NA | Yes | NA | NA | Yes | Fair |
| Sun et al. (2021)^27^ | Yes | Yes | CD | Yes | No | No | No | Yes | No | NA | Yes | NA | NA | Yes | Good |
| Thayer & Gildner (2020)^45^ | Yes | Yes | CD | Yes | No | No | No | Yes | No | NA | Yes | NA | NA | Yes | Good |
| Timmins et al. (2022)^54^ | Yes | Yes | Yes | Yes | No | No | No | No | No | NA | Yes | NA | NA | Yes | Poor |
| Trógoloet al. (2022)^90^ | Yes | Yes | CD | Yes | No | No | No | Yes | No | NA | Yes | NA | NA | No | Fair |
| Van de Velde et al. (2021)^91^ | Yes | Yes | NR | Yes | No | No | No | Yes | Yes | NA | Yes | NA | NA | Yes | Good |
| Wathelet et al. (2020)^92^ | Yes | Yes | No | Yes | No | No | No | No | No | NA | Yes | NA | NA | Yes | Good |
| Wathelet et al. (2021)^93^ | Yes | Yes | No | Yes | No | No | No | Yes | No | NA | Yes | NA | NA | Yes | Good |
| Wathelet et al. (2022)^94^ | Yes | Yes | No | Yes | No | No | No | Yes | No | NA | Yes | NA | NA | Yes | Good |
| Wichaidit et al. (2022)^95^ | Yes | Yes | Yes | Yes | Yes | No | No | NA | No | NA | Yes | NA | NA | Yes | Good |
| Zajacova et al. (2020)^37^ | Yes | No | No | Yes | No | No | No | No | No | NA | Yes | NA | NA | Yes | Fair |
| Zhao et al. (2021)^30^ | Yes | Yes | Yes | Yes | No | No | No | Yes | No | NA | Yes | NA | NA | Yes | Good |

*Note*: See key below for quality assessment questions. Given the subjective nature of the quality assessment tool, we suggest that the ratings (and particularly the overall ratings) be interpreted with caution.

Key:

1. Was the research question or objective in this paper clearly stated?

2. Was the study population clearly specified and defined?

3. Was the participation rate of eligible persons at least 50%?

4. Were all the subjects selected or recruited from the same or similar populations (including the same time period)? Were inclusion and exclusion criteria for being in the study prespecified and applied uniformly to all participants?

5. Was a sample size justification, power description, or variance and effect estimates provided?

6. For the analyses in this paper, were the exposure(s) of interest measured prior to the outcome(s) being measured?

7. Was the timeframe sufficient so that one could reasonably expect to see an association between exposure and outcome if it existed?

8. For exposures that can vary in amount or level, did the study examine different levels of the exposure as related to the outcome (e.g., categories of exposure, or exposure measured as continuous variable)?

9. Were the exposure measures (independent variables) clearly defined, valid, reliable, and implemented consistently across all study participants?

10. Was the exposure(s) assessed more than once over time?

11. Were the outcome measures (dependent variables) clearly defined, valid, reliable, and implemented consistently across all study participants?

12. Were the outcome assessors blinded to the exposure status of participants?

13. Was loss to follow-up after baseline 20% or less?

14. Were key potential confounding variables measured and adjusted statistically for their impact on the relationship between exposure(s) and outcome(s)?

15. Overall quality rating

Abbreviations: CD = cannot determine; NA = not applicable; NR = not reported; Pop. = population; Part. = participation

**Table S2**

*Quality Assessment Ratings for Longitudinal Studies*

|  | 1 | 2 | 3 | 4 | 5 | 6 | 7 | 8 | 9 | 10 | 11 | 12 | 13 | 14 | 15 |
| --- | --- | --- | --- | --- | --- | --- | --- | --- | --- | --- | --- | --- | --- | --- | --- |
| Badellino et al. (2022)^60^ | Yes | Yes | NR | No | No | No | Yes | Yes | Yes | No | Yes | NA | No | No | Poor |
| Baranov et al. (2022)^20^ | Yes | Yes | Yes | Yes | No | No | Yes | No | No | No | Yes | NA | Yes | Yes | Fair |
| Batterham et al. (2021)^61^ | Yes | Yes | NR | Yes | Yes | No | Yes | Yes | Yes | No | Yes | NA | No | Yes | Fair |
| Bierman et al. (2021)^61^ | Yes | Yes | NR | Yes | No | No | Yes | Yes | Yes | Yes | Yes | NA | No | Yes | Good |
| Canet-Juric et al. (2020)^55^ | Yes | Yes | NR | Yes | No | No | No | Yes | Yes | No | Yes | NA | NR | No | Poor |
| Choi et al. (2023)^21^ | Yes | Yes | NR | Yes | No | No | Yes | Yes | Yes | No | Yes | NA | NR | Yes | Good |
| Feter Et al. (2021)^24^ | Yes | Yes | NR | Yes | Yes | No | Yes | No | Yes | No | Yes | NA | Yes | No | Fair |
| Graupens-Berger et al. (2022)^62^ | Yes | Yes | Yes | Yes | No | No | Yes | Yes | Yes | Yes | Yes | Yes | Yes | Yes | Good |
| Hagen et al. (2023)^56^ | Yes | Yes | NR | No | No | No | Yes | No | Yes | No | Yes | NA | No | Yes | Poor |
| Hertz-Palmor et al. (2021) - Study 1^57^ | No | Yes | NR | Yes | No | No | Yes | Yes | Yes | No | Yes | NA | No | Yes | Fair |
| Hertz-Palmor et al. (2021) - Study 2^57^ | No | Yes | NR | Yes | No | No | Yes | Yes | Yes | No | Yes | NA | No | Yes | Fair |
| Lee et al. (2022)^22^ | Yes | Yes | Yes | Yes | No | No | Yes | Yes | Yes | No | Yes | NA | NR | Yes | Good |
| Murray et al. (2023)^19^ | Yes | Yes | Yes | Yes | No | No | Yes | Yes | Yes | No | Yes | NA | Yes | Yes | Good |
| Shuster et al. (2021)^58^ | Yes | Yes | NR | No | No | No | Yes | Yes | Yes | Yes | Yes | NA | No | Yes | Fair |
| Simonse et al. (2022)^1^ | Yes | Yes | NR | Yes | No | No | Yes | Yes | Yes | Yes | Yes | NA | No | Yes | Good |
| Strizzi et al. (2023)^59^ | Yes | Yes | No | Yes | No | No | Yes | Yes | Yes | No | Yes | NA | No | Yes | Fair |
| Weber et al. (2023)^63^ | Yes | Yes | NR | Yes | No | No | Yes | Yes | Yes | No | Yes | NA | No | Yes | Fair |

*Note*: See Table 1 note for quality assessment questions. Given the subjective nature of the quality assessment tool, we suggest that the ratings (and particularly the overall ratings) be interpreted with caution.

**Table S3.** *Data Extracted from Cross-Sectional Studies in General Population Samples*

| **Authors, date,**  **country** | **Study period / COVID-19 phase** | **Analytic sample** | **Mental health variable and measure** | **Financial change during COVID-19 variable and measure** | **Main (relevant) findings** |
| --- | --- | --- | --- | --- | --- |
| Allen et al. (2021)^68^, United Kingdom | 15 Apr - 8 Jun, 2020: First lockdown | General adult population (*N* = 200). Age 18-62; 7.86.0% female, 93.0% White, 92.5% UK-based, 73.5% students | **Psychological well-being:** Psychological well-being scale (PWB18)  **Anxiety:** Generalized Anxiety Disorder Scale (GAD-7) **Depression:** Patient health questionnaire (PHQ-9) **Loneliness:** UCLA loneliness scale (UCLA3) | **Financial changes:** "Are you facing reduced work hours and reduced income due to the COVID-19 pandemic?" (Yes, no or not applicable) | Those who had less income had poorer psychological wellbeing. People with less working hours/income reported significantly more anxiety, depression and loneliness. |
| Aruta (2021) ^28^, Phillipines | Last week of Apr to second week of May 2020: Early phase of community quarantine | General Filipino adult population (*N* = 401). Age 18-68 years (*M* = 30.09, *SD* = 11.15); 63.34% female, 36.41% male, 1 did not report gender | **Distress:** Brief Symptoms Inventory (BSI) - symptoms related to anxiety and depression | **Financial difficulties:** “Do you or your family currently experience financial difficulties due to the Coronavirus crisis (such as unemployment, reduced business activity and so on)?” With a Likert scale (1-5) | Those with more familial financial difficulties were more likely to experience higher psychological distress (β = .18, *t* = 4.00, *p* ≤ .001). |
| Aruta et al. (2021)^69^, Phillipines | Last week of Apr to second week of May 2020: Early phase of community quarantine | General Filipino adult population (*N* = 401). Age 18-68 years (*M* = 30.09, *SD* = 11.15); 63.34% female, 36.41% male, 1 did not report gender | **Psychological distress:** Brief Symptoms Inventory (BSI) - symptoms related to anxiety and depression **Quality of life:** My LifeToday scale (MLT) | **Financial difficulties:** “Do you or your family currently experience financial difficulties due to the Coronavirus crisis (such as unemployment, reduced business activity and so on)?” with a Likert scale (1-5) | Those with more financial difficulties had more psychological distress. Psychological distress mediated the negative relationship between financial difficulties and quality of life (*B* = −0.04, β = −0.06, SE = 0.02, p ≤ 0.001). |
| Chung et al. (2021)^34^, China | Sept 11 to Oct 12, 2020 | General adult population in Hong Kong (*N* = 1053). Age: 33.2% 18–39, 45.6% 40–64,  21.2% ≥65; 55.2% female | **Depression and Anxiety:** Patient Health Questionnaire (PHQ-4) | **Financial worry:**  1. Financial and livelihood worries (e.g., job or income loss), rated on a 5-point scale. 2. Worry about personal savings rated on a 5-point scale. | Financial and livelihood worries predicted poorer mental health (β=0.134; p=0.007). These worries mediated the association between greater deprivation and mental health disorders (β = 0.222 × 0.134 = 0.030; p = 0.004). |
| Clark-Deelder (2022)^32^, Uganda | Dec 2020 to Apr 2021: Peak of first wave in Dec 2020 | General Ugandan adult population (*N* = 4066) | **‘Psychological distress’:** Patient Health Questionnaire (PHQ-4) | **Financial change:** Change in income since March 2020 due to COVID-19 restrictions (4 response options for extent of change) | No clear association between psychological distress (anxiety and depression) and income change. |
| Dawel et al. (2020)^72^, Australia | Mar 28–31. 2020: First wave | General adult population (*N* = 1296). Age (*M* = 46, *SD* = 17.3). 50.2% female, 49.8% male | **Depression** Patient Health Questionnaire-9 (PHQ-9)  **Anxiety**  Generalized Anxiety Disorder-7 (GAD-7)  **General psychological wellbeing**  World Health Organization Wellbeing Index (WHO-5) | **Income loss:** Job loss due to COVID-19" (yes or no) **Financial distress:** Financial distress due to COVID-19 (6 point scale) | COVID-19 induced financial distress predicted greater depression and anxiety and less general psychological wellbeing. |
| Elaidy et al. (2023)^73^, Kuwait | Nov 2020 - Jan 2021 (exact dates not reported) | General adult population (*N* = 415). Age (*M* = 35.03, *SD* = 10.9) years, 63.1% female | **Distress:** Arabic version of Kessler Psychological Distress Scale (K-10) | **Financial change:** COVID-19 impacted financial situation (yes or no) | Psychological distress was greater for those whose financial situation was affected by COVID-19 (OR = 1.184, 95% CI 1.040, 1.348). |
| Ertl et al. (2022)^75^, 59 countries | April 19 to May 3, 2020: Early phase; effects of pandemic different in each country though most had peak cases and restrictions | General adult population (*N* = 6882). Age: 18-94, (*M* = 42.30 years, *SD* = 13.95); 78.8% female, 20.9% male, 0.2% transgender or nonbinary | **Trauma-Related Distress:** Child-Revised Impact of Events Scale (CRIES-8) posttraumatic intrusion/avoidance | **Financial changes and difficulties:** Epidemic-Pandemic Impacts Inventory (EPII), 9 items: 1. 7 questions from Work and Employment subscale including, "Laid off from job or had to close own business" 2. 2 questions from Economic subscale including, “Unable to get enough food or healthy food”) | Job loss (β = 0.029, *p* = 0.022), reduction of work hours (β = 0.042, *p* = <.001) and inability to pay bills (β = 0.045, *p* = 0.001) predicted trauma-related distress. |
| Frankenthal et al. (2023)^76^, Israel | May - Sept, 2020. T1: May – Jun 2020 (reduction of cases and restrictions) T2: July – Sept 2020 (rising cases and restrictions) | General adult population (*N* = 2504). Age: 31.8% 21-34; 29.8%35-49, 22.3% 50–64, 16.1% 65+; 51.1% female, 48.9% male | **Depression and Anxiety:** 5-item Mental Health Inventory | **Financial changes:** Change in average monthly household income (declined or no change/increased) | A decrease in average household income per month during COVID-19 was associated with poor mental health (i.e., greater anxiety and depression) |
| Guerrero and Barnes (2022)^31^, Canada | T1: fall of 2020 (second COVID wave); T2: spring of 2021 (third wave began in Mar with more restrictions). Exact dates not recorded. | General adult population (*N* = 22,721). Age 18+ years - overall age and gender not reported (reported by mental health profile in Table 2 of article) | **Anxiety:** Generalized Anxiety Disorder Scale (GAD-7)  **Depression:** Patient Health Questionnaire-9 (PHQ-9)  **Distress:** Kessler Psychological Distress Scale (K10) | **Financial changes:** Loss of job or income **Financial difficulty:** Difficulty meeting financial obligations/ essential needs. | Those with low/moderate and severe mental health difficulties (including anxiety, depression, and distress) had increased odds of struggling to meet financial obligations and losing their job/income compared to those without mental health difficulties. |
| Hyland et al. (2020)^78^, Ireland | Mar 31 and Apr 5, 2020: first week of quarantine (31 days post first COVID-19 case in Ireland; 19 days after initial social distancing restrictions began). | General adult population (*N*=1041). Age 18-88 (*M* = 44.97, *SD* = 15.76). 51.5% female, 48.2% male, 0.3% transgender or prefer not to say | **Depression:** Patient Health Questionnaire-9 (PHQ-9) **Anxiety:** Generalized Anxiety Disorder 7-item Scale (GAD-7) | **Financial changes:** Loss of income due to COVID-19 (yes, no or unsure) | Income loss due to COVID-19 was positively associated with screening positive for anxiety and depression. |
| Kim (2021)^38^, United States | Sept 2—Dec 21, 2020: Rise in infection rates with some restrictions in place | General adult population (*N* = 91,222). Age 18-64 (*M* = 40.3); 51.9% female | **Anxiety and depression:** Patient Health Questionnaire (PHQ-2) | **Financial hardship:** Difficulty paying expenses (not at all/a little/somewhat/very difficult) | Those who reported experiencing financial hardship 'somewhat' had twice as greater risk of experiencing anxiety (1.96, 95% CI = 1.85–2.08, *p* < .001) and depression (2.75, 95% CI = 2.54–2.98, *p* < .001) than those who reported no hardship. Those who reported 'considerable' financial hardship had 3 times greater risk of experiencing anxiety (2.61, 95%CI = 2.46–2.76, *p* <.001) and depressive symptoms (2.75, 95%CI = 2.54–2.98, *p* <.001) than no hardship. |
| Lu et al. (2020)^81^, China | Jun 8-18, 2020: Two months since lockdown eased; few cases of COVID | Adults in Wuhan (N = 1417): 73% general public; 27% frontline healthcare workers; 70% aged 26-40. 83.4% female, 16.6% male | **Depression:** Patient Health Questionnaire (PHQ-9) **Anxiety:**  Generalised Anxiety Disorder scale (GAD-7) **Post-Traumatic Stress Disorder:**  Posttraumatic Stress Disorder Checklist (PCL) | **Financial changes:** Income change during the COVID-19 outbreak (increase, no change or decrease) | Those with income loss were at more risk of anxiety (OR [95%CI]: 2.49 [1.43–4.33]; *p* = 0.001) and depression (OR [95%CI]:1.85 [1.07–3.21]; *p* = 0.028). |
| Mougharbel et al. (2021)^83^, Canada | May 8-12, 2020: First wave (peak of first wave was end of May) | English-speaking Canadian adults (*N* = 1005); 504 male, 498 female, 3 other; 698 white | **Depression:** Centre for Epidemiologic Studies Depression Scale 3-item (CES-D)  **Anxiety:** Generalized Anxiety Disorder scale (GAD-7) | **Financial worry:** Worries about the impact of COVID-19 on personal financial situation (very, somewhat, not very, not at all) | Financial worries were associated with greater likelihood of anxiety and depression. Binge drinking and anxiety (OR 3.09; 95% CI, 1.97–4.85), binge drinking and depression (OR 1.81; 95% CI, 1.12–2.94). |
| Nagasu et al. (2021)^36^, Japan | Mar 26-28, 2020: Early phase | General adult population (*N* =11,342). Age 20-64 (*M* = 43.5, *SD* = 12.0); 50.6% men, 49.4% women | **Psychological Distress:** Japanese version of Kessler Psychological Distress scale (K6) | **Financial worry:** Worry about impact on finances (e.g., income) after COVID-19 (yes or no) | There was a non-significant trend for a positive association between worries about the impact of COVID-19 on finances with psychological distress (Adjusted OR 1.084, 95% Cl[0.941-1.191]). |
| Nelson et al. (2020)^84^, Unites States, Canada, and Europe | Mar 19-Apr 10, 2020: Early phase | General adult population (*N* = 2065). Age 18-77 (*M* = 34.40, *SD* = 11.49); 69.20% female | **Anxiety:** Generalized Disorder Scale (GAD-2)  **Depression:** Patient Health Questionnaire-2 (PHQ-2). | **Financial strain:** 1. "Have you lost income?" (yes or no) 2. "Money situation" (comfortable, enough, cutting back, not enough) 3. "Job loss" (yes or no) 4. "Food security: ran out and didn't have money to get more" (never, sometimes, often) | There was a positive association between employment loss and depressive symptoms (B = 0.456, SE = 0.084, *p* < 0.001, 95% CI [0.291 – 0.622]) and anxiety (B = 0.346, SE = 0.093, *p* < 0.001, 95% CI [0.165 – 0.528]). |
| Odani et al. (2022)^85^, Japan | August 25 - September 30th, 2020: Early phase | General population (*N* = 2065). Age 15-79; 50.3% female, 49.7% male | **Psychological Distress:** Kessler 6 scale (K6) | **Financial hardship:** 1. Income change since COVID-19 began (scale 0-100) 2. If they had a shortage of money for necessities  3. Whether the hardships only began after COVID-19 started **Financial anxiety:** 1. If they felt anxious regarding the budget for their household | Income loss was associated with experiencing serious psychological distress. Shortage of money and anxiety about finances were significantly associated with serious psychological distress. |
| Rahman et al. (2020)^33^, Australia | Jun 1-30, 2020: During strict lockdown restrictions | General adult population (*N* = 587). Age 18-77 (*M* = 41.3, *SD* = 12.5); 61.8% female | **Distress:** Kessler Psychological Distress Scale (K10) | **Financial change:** "Covid-19 impacted financial situation" (yes or no) | Having a financial situation impacted by COVID-19 was not associated with having psychological distress (adjusted OR 1.14, 95% Cls 0.76-1.72). |
| Sekscinska et al. (2022)^88^, Poland | May 5-12 2020: First wave | General adult population (*N* = 977). Age 18-87 (*M* = 38.68, *SD* = 11.59); 64.5% female | **Anxiety:** Polish version of the General Anxiety Disorder scale (GAD-7) **Depression:** Polish version of the Patient Health Questionnaire (PHQ-9) | **Objective financial situation:** Income, ownership of savings, whether they saved money pre COVID-19, financial liabilities. **Subjective financial situation:** Rated: financial situation, COVID-19-related financial change, financial security, and job security. | Objective and subjective financial difficulties predicted greater anxiety and depression. Higher financial security associated with reduced anxiety and depression.  Objective financial variables: anxiety (R2 change = 0.006, *p* = 0.235); depression (R2 change = 0.005, *p* = 0.308). Subjective financial variables: anxiety (R2 change = 0.029, *p* < 0.001); depression (R2 change = 0.027, *p* < 0.001). |
| Shevlin et al. (2020)^89^, United Kingdom | March 23 and 28 2020: First wave - study began on same day that lockdown was announced | General adult population (*N* = 2025). Age 18-83 (*M* = 45.55, *SD* = 15.9); 51.7% female, 48.0% male, 0.3% transgender/prefer not to say/other | **Depression:** Patient Health Questionnaire (PHQ-9)  **Anxiety:** Generalized Anxiety Disorder (GAD-7) **Traumatic stress:** International Trauma Questionnaire (ITQ) ICD-11 PTSD | **Income change:** Household income lost due to COVID-19 (yes, no, not sure) | Those who had a reduction of income during COVID-19 had greater risk of depression and anxiety. Income reduction had little effect on traumatic stress. |
| Shevlin et al. (2022)^29^, United Kingdom | Aug 6 - Sept 28, 2021: Wave 6 - only some social distancing restrictions in place | General adult population (*N* = 2025). Age 18-83 (*M* = 45.55, *SD* = 15.9); 51.9% female, 47.8% male, 0.2% transgender/prefer not to say/other | **Generalised Anxiety Disorder:** Generalised Anxiety Disorder scale (GAD-7)  **Major Depressive Disorder:** Patient Health Questionnaire (PHQ-9) | **Debt changes:** 1. "Has your overall debt increased or decreased this month due to COVID-19" 2. Subjective rating of the manageability of their debt on Likert scale (1-5) | Dose-response association with greater debt management issues and greater depression and anxiety.   Adjusted odd ratios anxiety: 2.28-11.18 from some debt problems to very serious. Adjusted odd ratios depression: 2.8-16.21 from some debt problems to very serious. |
| Shuster et al. (2021)^58^, United States | Apr 2 - Jun 4, 2020: First wave - some restrictions | General adult population (*N* = 743). T1 Age (*M* = 35.04, *SD* = 13.08); 49.18% females | **Depression:** Zung Self-Rating Depression scale (SDS) **Anxiety:** State-Trait Anxiety Inventory (STAI) | **Economic impact:** "Rate the impact that COVID-19 had on your financial situation" | Lower income and worsening financial situation due to COVID-19 was associated with greater depression and anxiety. |
| Simha et al. (2020)^35^, United Kingdom | April 23 and 28, 2020: First wave | Clinically high risk and financially vulnerable participants (*N* = 15, 691). Age (M = 51.2); 58.4% females. 21% financially vulnerable | **Mental Distress:**  General Health Questionnaire (GHQ-12) | **Financial Vulnerability:** How financially vulnerable participants thought they were (very low, low, medium, high, extreme vulnerability) | Participants who thought they were financially vulnerable due to COVID-19 had greater mental distress (β = 0.294, 95% CI, LLCI = 1.88, ULCI = 2.09). |
| Wichaidt et al. (2022)^95^, Thailand | Late Apr 2021: Beginning of third wave | General adult population (*N* = 1555). Age (*M* = 41.0, *SD* = 0.3). 51.7% female, 48.3% male | **Anxiety:** Generalised Anxiety Disorder Scale (GAD-7) Thai version **Depression:** Patient Health Questionnaire (PHQ-2) Thai version | **Emergency cash reserves:** How participants would cover an emergency cash payment within one week (responses selected from a list of options) **Economic Distress:** Economic distress since COVID-19 and economic distress in previous 30 days | Anxiety and depression were higher in those who experienced economic distress within last 30 days than those who did not (the effect for depression was rendered non-significant when demographic characteristics were covaried). Having emergency cash reserves did not moderate the association between economic distress and anxiety and depression. |
| Zajacova et al. (2020)^37^, Canada | Survey 1: Mar 29 - Apr 3, 2020. Survey 2: May 4 - May 10, 2020: Early phase with distancing restrictions | General population (*N* = 4627 in Mar, 4600 in May): Age 15+ years: 14.2% 15-24, 16.9% 25-34, 16.1% 35-44, 15.2% 45-54, 16.7% 55-64, 14.9% 65-74, 6.1% 75+; 51.7% female, 48.3% male | **Anxiety:**  Generalized Anxiety Disorder Scale (GAD-7) (Cycle 2 only) | **Employment security: "**I might lose my main job or main self-employment income sources in the next four weeks" (1-5 agreement scale) **Financial hardship:** Impact of COVID-19 on meeting financial obligations (major, moderate, minor, none, cannot tell) **Food security:** Food secure or insecure (Cycle 2 only) | Greater financial hardship due to COVID-19, food insecurity, and unsecure employment status were associated with greater anxiety. |
| Zhao et al. (2021)^30^, China | Apr 9-23, 2020: 2–4 weeks after second wave peaked | General Chinese adult population in Hong Kong (*N* = 1501). Age (*M* = 41.3); 55.2% female, 44.8% male | **Stress:** Perceived Stress Scale (PSS-4) **Anxiety and depression:** Patient Health Questionnaire (PHQ-4) including 2 questions from General Anxiety Disorder scale (GAD-2) | **Income loss:** Income reduction since the start of COVID-19 (none, small, half, larger or unemployed) | Income loss due to COVID-19 was positively associated with stress, anxiety, and depression; these mental health problems were further increased for those who lost their jobs during the pandemic. |

**Table S4.** *Data Extracted from Cross-Sectional Studies in Student Samples*

| **Authors, date, country** | **Study period / COVID-19 phase** | **Analytic sample** | **Mental health variable and measure** | **Financial change during COVID-19 variable and measure** | **Main (relevant) findings** |
| --- | --- | --- | --- | --- | --- |
| Davis et al. (2021)^25^, Liberia | Jul 1 - Oct 31, 2020: During first (Mar -Sept 20) and second (Sept 20-May 21) waves | Medical/pharmacy students (*N* = 113). Median age 28 years (IQR = 26, 32). 61.9% men, 75.2% single | **Depression:** Patient Health Questionnaire - Depression Scale (PHQ-8) | **COVID-19-related financial concerns:** Series of questions about how finances were impacted by COVID-19 (range of response options varying per question) | Worries about finances were associated with increased stress, distress, and depression. Variable depression scores in those who reported actual income loss. |
| ElTohamy et al. (2022)^74^, United States | Jan - early Jun, 2021 (most data collected in Feb and Mar): Wave of COVID-19 variant ‘alpha’ (some social distancing restrictions; rise of cases through winter and spring 2021) | Undergraduate students (*N* = 65,568). Age 87.3% 18-24 years; 68.2% female | **Psychological Distress:** Kessler Screening Scale for Psychological Distress  **Anxiety:** Asked if ever diagnosed by professional **Depression:** Asked if ever diagnosed by professional | **Financial hardship:** Effect of the COVID-19 pandemic on financial situation - rated on a 5-point scale from 'a lot more stressful' to 'a lot less stressful'. | Those with greater financial hardship due to the pandemic had the greatest chances of experiencing psychological distress (OR: 1.78, *p* < 0.0001). |
| Galanza et al. (2023)^26^, Phillipines | Sept - Oct, 2020 (exact dates not reported) | Filipino university students (*N* = 681). Age 18-24 years (*M* = 19.45, *SD* = 1.13); 64.90% female | **Depression, Anxiety, Stress:** Depression Anxiety Stress Scale (DASS-21) | **Financial difficulties:** “Do you or your family currently experience financial difficulties due to the Coronavirus crisis?” (yes/no) | Financial difficulties predicted greater depression and anxiety (though not stress). |
| Irfan et al. (2021)^79^, Malaysia | Jun - Jul, 2020: few cases during this time | University students (N = 958). Age 69.6% below 20 years; 70.9% female, 29.1% male. | **Anxiety:** Generalised Anxiety Disorder Scale (GAD-7) | **Income change:** Family income decreased (yes/no) | Reduction in family income due to COVID-19 increased odds of greater anxiety by 1.7 (95% CI = 1.34 to 2.17). Loss of family income was one of the strongest risks for experiencing greater anxiety. |
| Sato et al. (2023)^87^, Japan | Aug 25 - Sept 30, 2020: Wave 2 (Aug -Sept 2020) where transmission was increasing | Undergraduate students (*N* = 958) median age 20; 56.8% women | **Distress:** Kessler Psychological Distress Scale (K6) | **Income change:** 1. Series of questions including income change (response as percentage lost), job loss (yes or no), unpaid wages (yes or no) 2. Cannot afford necessities, school fees or food (yes, no or started before pandemic) | Reduction in income was associated with psychological distress. Compared to those whose income did not change during COVID-19, those whose income decreased by 50-99% reported greater distress (prevalence ratio [PR] = 1.48). Distress in those with unpaid wages compared to paid wages: PR = 1.44. Distress in those with money shortage for necessities compared to no shortage: PR = 1.45. |
| Sun et al. (2021)^27^, China | Mar 20 - Apr 10, 2020: Early in quarantine (two months after outbreak began) | University students (*N* = 1912). Age (*M* = 20.28, *SD* = 2.10); 69.77% female | **Anxiety:** Generalized Anxiety Disorder Scale (GAD-7)  **Depression:** Patient Health Questionnaire (PHQ-9) **Traumatic stress:** Impact of Events scale (IES): questions asked in context of COVID-19 | **Financial stress:** Amount of financial stress caused by COVID-19 (5-point scale) | Financial stress due to COVID-19 predicted increased anxiety, depression, and traumatic stress. |
| Van de Velde et al. (2021)^91^, 26 high/middle income countries | Apr 27 - July, 2020: First wave with lockdown across all countries | Higher education students (*N* = 20, 103; 73.9% female; 78.2% less than 26 | **Depression:** Center for Epidemiologic Studies (CES-D-8) questionnaire | **Financial status:** 1. Had enough money for monthly costs before COVID-19 (struggled or didn't) 2. Change in financial situation (worse or better during COVID-19) 3. How many people they can borrow money from (up to 5 or more) | Those with financial issues before COVID-19, those with no one or only 1-2 people to borrow money from, and those with more financial difficulty since COVID-19 had greater depression. |
| Wathelet et al. (2020)^92^, France | Apr 17 - May 4, 2020: During lockdown in the acute phase | University students (*N* = 69,054). Age (median 20 years); 72.8% female, 26.1% male, 1.1% nonbinary | **Distress:** Impact of Events Scale - Revised (IES-R) **Stress:** Perceived Stress Scale (PSS-10) **Anxiety:** State-Trait Anxiety Inventory (STAIY-2)  **Depression:** Beck Depression Inventory (BDI-13)  **Suicidal thoughts** in preceding month (yes or no; unstandardised question) | **Financial change:** Income loss due to COVID-19 | Income loss was associated with distress, stress, anxiety, depression, and suicidal thoughts. Compared to those who did not lose income, those who did were at greater risk of experiencing at least one mental health problem (OR, 1.28; 95%CI, 1.22-1.33; P < .001). |
| Wathelet et al. (2021)^93^, France | Jun 15 - Jul 15, 2020: One month after the end of the first lockdown | University students (*N* = 22,883). Female (72.2%), male (25.8%), others (1.5%); average age 21 (+4). | **Post Traumatic Stress Disorder (PTSD):** PTSD Checklist (PCL-5) | **Financial change:** Income loss due to COVID-19 | Income loss associated with probable PTSD (OR =1.20 [95% CI: 1.09–1.31], *p* < 0.001). |
| Wathelet et al. (2022)^94^, France | Jul 21 - Aug 31, 2021: 15 months after the start of the pandemic | University students (*N* = 44,898). Age 18-21 years (median = 19) Female (70.7%), men (27.7%), nonbinary (1.6% and were excluded). | **Post Traumatic Stress Disorder (PTSD):** PTSD Checklist (PCL-5) **Stress:** Perceived Stress Scale (PSS-10) **Depression:** Beck Depression Inventory (BDI-13) **Anxiety:** State-Trait Anxiety Inventory State (STAI Y-2) **Suicidal thoughts** in preceding month (yes or no; unstandardised question) | **Financial difficulties:** Difficulty making ends meet each month (significant, moderate, none) | Greater financial difficulties were associated with greater PTSD, stress, depression, anxiety, and suicidality. Relative to those with no/few financial difficulties, those with moderate difficulties had ORs ranging from 1.36 (95% CI, 1.27-1.45) for suicidal thoughts to 1.75 (95% CI, 1.67-1.84) for PTSD. Those with significant difficulties had ORs ranging from 2.19 (95% CI, 2.03-2.35) for suicidal thoughts to 3.44 (95% CI, 3.21-3.68) for depression. |

**Table S5.** *Data Extracted from Cross-Sectional Studies in Other, Specific Samples*

| **Authors, date, country** | **Study period / COVID-19 phase** | **Analytic sample** | **Mental health variable and measure** | **Financial change during COVID-19 variable and measure** | **Main (relevant) findings** |
| --- | --- | --- | --- | --- | --- |
|  |  |  |  |  |  |
| Bryson et al. (2021)^46^, Australia | 6 May - 23 Nov 2020: first wave ended by May, second wave not begun though ended in Oct with some restrictions | Mothers and children with adversity pre-COVID (*N* = 319); mothers' ages not reported, all female | **Maternal Mental Health:**  Depression, Anxiety and Stress Scales (DASS-21) **Child Mental Health:** Coronavirus Health Impact Survey 3.0 Child Mood States Scale (CRISIS 3.0) | Questions from Household, Income and Labour Dynamics in Australia: **Changes to financial circumstances:** Has there been a job loss (yes or no) or a reduced ability to work? (yes or no) **Financial harship:**  Struggle to pay rent, bills, food, healthcare or car insurance (yes or no) | More financial hardship was associated greater maternal and child mental health symptoms even after pre-COVID-19 mental health was controlled (β = 0.27, *p*<0.001). |
| Bui et al. (2021)^47^, United States | 23 Apr 2020 surveys began: First wave (Mar-Sept 2020) - data pooled into cross-sectional dataset | Older adults (*N* = 94,550). Age: 55-88; 55.2% female; 80,5233 non-Hispanic white, 5,718 non-Hispanic black, 2,908 non-Hispanic Asian, 5371 Latino | **Emotional distress:** 4 questions from Patient Health Questionnaire and Generalized Anxiety Disorder Scale. | **Financial hardship:** 1. Has anyone in your household suffered a loss of employment income since March 13 (yes or no) 2. Did your household make the last rent or mortgage payment on time (did or didn't) | Those with income loss who expected further losses experienced greater distress.  Those who did not make a house payment on time and didn't anticipate being able to experienced greater distress. |
| Coulombe et al. (2020)^71^, Canada | Mar 2020: First wave (one week after social distancing measure) | Canadian workers (*N* = 1122) who had worked at least 20hrs per week pre-COVID. Age (*M* = 39.43, *SD* = 12.13); 74.2% female; 85.5% born in Canada; 89% Caucasian | **Stress:** Perceived Stress Scale (PSS) to assess perceived stress within the last week **Distress:** Patient Health Questionnaire (PHQ-4) for anxiety and depression | **Income Loss:** Report percentage of current income compared to pre-pandemic (scale of 0-100) | Income loss was associated with greater stress and distress. |
| García-Fernandez et al. (2020)^48^, Spain | Mar 29 - Apr 5, 2020: First wave: Peak of the SARSCoV-2 infection in Spain | Older adults (*N* = 1639). 150 were aged 60 years or over and 1489 were under 60 | **Anxiety:** Hamilton Anxiety Scale (HARS) **Depression:** Beck Depression Inventory (BDI) | **Income loss: "**Economic losses" (yes or no) | Those who experienced financial losses had more anxiety (*F*(1, 146) = 6.3, *p* = 0.013, hp 2 = 0.04) and depression (*F*(1, 146) = 4.2, *p* = 0.04, hp 2 = 0.03) than those who did not. |
| Halim et al. (2022)^52^, Bangladesh | Jan 5 - Feb 25, 2021: before start of second wave (which began in Mar), low no. of cases | Middle income participants (*N* = 150). Age (*M* = 40.73, *SD* = 10.07); 86% male | **Depression:** Patient Health Questionnaire (PHQ-9) **Anxiety:** Generalized Anxiety Disorder (GAD-7) | **Financial stress:** 4 questions about facing economic stress, poverty level since COVID-19, responsibility to financially support family and family budget **Income change:** 3 questions about income changes due to COVID-19, the amount of income change and household debt | Most had moderate depression and anxiety symptoms due to financial problems. Depressive and anxiety symptoms were greater among those with income loss and debt. Income loss due to COVID-19 increased anxiety by a small point scale (0.00, *p* < .10). Income loss reduced depression slightly (-0.00, p < .05). |
| Haque et al. (2022)^77^ | Jun 2020: large no. of cases with lockdown restrictions | Informal waste workers (*N* = 176). 75.6% aged 26-50; 55.0% male, 45% female | **Psychological Distress:** General Health Questionnaire (GHQ-12) | **Income change:** Whether income has reduced since COVID-19 based on household income | Income loss due to COVID-19 increased risk of psychological distress (RR: 1.60, 95% CI: 1.06–2.41). |
| Islam et al. (2021)^53^ | Aug - Sept, 2020: Restrictions lifted Sept 1 but cases were not particularly low | Impoverished urban Bangladeshi dwellers (*N* = 435). Age 18-85 (*M* = 45.0, *SD* = 12); 54.7% male | **Depression:** Patient Health Questionnaire (PHQ-9) - Bangla version **PTSD:** National Stressful Events Survey for PTSD - Short Scale (NSESSS-PTSD) - Bangla version | **Financial changes:** 1. "How has your family's monthly income changed due ot the impact of COVID-19?" (decreased, increased, unchanged) 2. Job loss due to COVID-19 (yes or no) | Income loss did not impact PTSD and depression, though having a household income of less than 10,000 BDT per month was linked to greater PTSD severity. |
| Lee (2022)^49^, 27 European countries | Apr 9-30, 2020: During the beginning of the outbreak | Middle- and older-adults aged 50 years or above (*N* = 31,757). Age (*M* = 59.99, *SD* = 7.03); 26.2% male, 73.8% female | **Mental health**: World Health Organization—Five Well-Being Index (WHO-5) | **Financial impact**: 2 items - perceived likelihood of losing job in next 3 months and having to leave current accommodation in next 6 months due to costs (5-point scale from *very likely* to *very unlikely*).  **Financial changes**: 2 items: whether household financial situation changed compared to 3 months ago (3-point scale of *became* *worse*, *remained* *same*, *became* *better*) and how well expenses cover necessary expenses (6-point scale of difficulty level) | Job security, less change in finances, and ability to make ends meet were all associated with better mental health, even when covarying demographic characteristics. |
| Lewis et al. (2022)^42^, United Kingdom | Jun - Aug, 2020: After first wave (approx. end May); some restrictions lifted, lower cases than during peak | Participants with history of mental illness (*N* = 2869). Age 18-94 (69.2% ≥35 or over, 30.4% <35); 77.6% female, 0.7% transgender, 20.1% male, 39% gender variant/non-conforming/non-binary; 95% white | **Anxiety:** Generalised Anxiety Disorder  **Depression:** Patient Health Questionnaire (PHQ-9)  **Psychological well-being:** Word Health Organization Well-Being Index (WHO-5) | **Financial impact:** Work, study or employment status change (yes or no) | Having a low income and income affected by the COVID-19 pandemic was associated with poorer mental health during the pandemic, including greater anxiety and depression and reduced wellbeing. |
| Li et al. (2023)^50^, 6 countries (China, Italy, Lithuania, Portugal, Slovenia, and United States) | Jul - Sept, 2020 (exact dates not recorded): small no. of cases in China. First wave in American and European countries | Young adults (*N* = 1679). Age 18–30 (*M* = 23.48, *SD* = 3.49); 20.8% male, 78.2% female, 1% gender queer | **Wellbeing:** Brief Inventory of Thriving (BIT-10) | **Financial changes:** 1. "I have lost job-related income due to the coronavirus" (5-point scale from *completely not true* to *completely true*) 2. Job loss due to COVID-19 | Income loss and job loss negatively correlated with psychological wellbeing.  Job loss negatively predicted current financial wellbeing but did not predict future financial wellbeing or psychological wellbeing. Income loss negatively predicted present and future financial wellbeing but did not predict psychological wellbeing. |
| Li et al. (2020)^80^, China | 25 Apr - 9 May, 2020: Strict restrictions, lower no. of cases than in initial outbreak | Chinese workers with COVID-related income loss (*N* = 398). Age (68.3% aged 26-40); 50.5% male, 49.5% female | **Depression:** Patient Health Questionnaire (PHQ-9) **Anxiety:** Generalized Anxiety Disorder (GAD-7) **Insomnia:** Insomnia Severity Index (ISI-7)  **Distress:** Revised Impact of Event Scale (IES-7) Chinese versions of tools used. | **Income loss:** Loss of income caused by COVID-19 (light >0 to 25%, middle 25–50%, or heavy >50%) | Those whose income was heavily impacted reported high levels of depression, anxiety, and insomnia. Adjusted odds of severe depression, anxiety, and distress symptoms were greater for those with larger income losses. |
| Liu et al. (2021)^44^, United States | May 6-8, 2020: Early acute phase, wave 1 | Pregnant women (*N* = 715), ≥8 weeks’ pregnant, aged 18-44 years, residing in and with prenatal care status in the US. Age: 91.5% aged 25 to 35; all female | **Postnatal Depression:** Edinburgh Postnatal Depression Scale (EPDS-10)  **Anxiety:** Generalized Anxiety Disorder Scale (GAD-7) | **Income loss:** Losing a source of income due to COVID-19 | Losing income due to COVID-19 was associated with greater odds of experiencing depression (adjusted OR 1.2 [0.5-3.0]) and anxiety (adjusted OR 1.7 [0.7-4.1]). |
| Miquel et al. (2022)^82^, Spain | Jun 2020 (exact dates not recorded): Directly after the first lockdown | Employees (*N* = 2381). Age (*M* = 43.0, *SD* = 11.8); 47,48% female, 52.53% male | **Depression:** Patient Health Questionnaire (PHQ-8) **Anxiety:** General Anxiety Disorder Scale (GAD-7)  **PTSD:** DSM-5 Checklist (PCL-5) **Panic attacks:** World Mental Health-International College Student (WMH-ICS) **Substance use disorders:** CAGE-AID questionnaire  **Suicide severity:** Columbia Suicide Severity Rating Scale (C-SSRS) | **Income change:** 1. "Are you unemployed or temporarily laid off due to the coronavirus pandemic?" 2. "Did you experience a significant loss of personal or family economic income due to the coronavirus pandemic?" **Perceived financial stress:** Adapted version of the Peri Life Events Scale | Those with job loss were more likely to have depression (OR = 1.48, 95% CI = 1.12–1.95). Those with income loss were more likely to have depression (OR = 1.30, 95% CI = 1.00–1.69).  Those with job and income loss had greater risk for PTSD. Those with income loss had increased risk for panic attacks. Job and income loss were not associated with generalised anxiety disorder and substance use disorder. |
| Patwary et al. (2022)^41^, Bangladesh | 17 Apr - 1 May, 2020: Early phase - beginning of lockdown | Students and working professionals (*N* = 744). Age: 94% 30 years or below; 58% male, 42% female | **Anxiety:**  Generalized Anxiety Disorder (GAD-7) **Stress:** Perceived Stress Scale-4 (PSS-4) | **Financial hardship:**  "Are you worried about the financial condition of your family during COVID-19?" (yes or no) | Financial hardship during the pandemic was positively associated with anxiety and predicted clinically significant anxiety levels in both students and workers. In students, but not workers, having financial hardship during the lockdown (vs. not) led to nearly twice the risk of greater anxiety levels (OR = 1.84, 95% CI = 1.11–3.05, *p* < 0.05). |
| Ruengorn et al. (2021)^86^, Thailand | Apr 21 - May 4, 2020: Early phase / wave 1 | Workers aged 18+ (*N* = 2303). Age (*M* = 34.5, *SD* = 10.2 years); 60% female, 37% male, 3% other | **Depression:** Patient Health Questionnaire (PHQ‐9) **Anxiety:** Generalised Anxiety Disorder Scale (GAD‐7) **Stress:** Percieved Stress Scale (PSS‐10) | **Economic burden:** 1. Job loss (yes or no) 2. Income loss (yes or no) 3. Financial problems (yes or no) | Job loss, income loss, and self-reported financial problems were associated with having depression, anxiety, and stress. Job loss increased odds of all mental health outcomes but the effect on depression was diluted when controlling covariates. Income loss (50% or less) increased odds of depression; income loss (50% or more) increased odds of anxiety; income loss did not predict stress. |
| Senturk et al. (2021)^39^, Turkey | Oct 25 - Dec 24, 2020: During lockdown; Dec included highest no. of cases and hospitalisations | Remote workers (*N* = 459). Age 24-60 (*M* = 35.64, *SD* = 6.8); 55.3% male, 44.7% female | **Depression, Anxiety, Stress:** Depression Anxiety Stress Scales (DASS-21) | **Financial state:** "How would you rate your financial situation in these days?" (0-10) **Financial concern:** "What is your financial expectation six months from now?" (0-10) | Financial concern predicted stress (β = –0.100, *p* = 0.040), though current financial state did not (β = -0.034, *p* = 0.482). No significant effect of current financial state and financial concern on depression and anxiety. |
| Sharma et al. (2022)^51^, Nepal | Jan 2021: End of the first wave (low cases) | Young adults (*N* = 1229). Age 18-35 (*M* = 25.0, *SD* = 4.1); 54.5% female | **Depression:** Patient Health Questionnaire-9 (PHQ-9) **Anxiety:** Generalized Anxiety Disorder-7 (GAD-7)  **PTSD:** PTSD checklist (PCL-C) | **Financial impact:** "Decrease in family income during COVID" (yes or no) **Financial worry:** "Worry about economic influences" (yes or no) | Income loss was positively associated with anxiety and PTSD, but not depression. Having 3 or more COVID-19 related stressors (including financial worry as one stressor) increased risk of depression and PTSD. |
| Spiro et al. (2021)^40^, United Kingdom | Apr 1 - Jun 15, 2020: First lockdown | Workers, performing arts professionals (*N* = 385). Age 18-86 (M = 44.08, SD = 13.9; 63% female | **Mental Wellbeing:** Mental Health Continuum – Short Form (MHC-SF) **Depression:** Center for Epidemiologic Studies Depression Scale (CES-D)  **Social Connectedness:** 15-Item Social Connectedness Scale **Loneliness:** 3-Item Loneliness Scale | **Financial hardship:** "Do you consider yourself to be in financial hardship as a result of the current public health situation?" (*no, a little, a lot*) | Perceived financial hardship was associated with worse mental wellbeing (B = -1.28, *p* = 0.006), depression (B = 0.18, *p* = 0.030), and loneliness (B = 0.13, *p* = 0.024). |
| Sujan et al. (2022)^43^, Bangladesh | Nov 2020 - Jan 2021: Before second wave in March 2021 (lower cases) | Adults with underlying health conditions (*N* = 971). Age (*M* = 42.29, *SD* = 15.86); 50.5% male, 49.95% female | **Depression:** Patient Health Questionnaire (PHQ-9)  **Anxiety:** Generalized Anxiety Disorder (GAD-7)  **Loneliness:** UCLA loneliness scale | **Financial hardship:** 1. Lost job (yes or no) 2. Financial difficulties (yes or no) 3. Eleven questions about managing the cost of medical treatment including being in debt, borrowing money, and receiving help from organisations to cover medical expenses | Those who reported poor health were 5x more likely to have anxiety and depression, and 3x more likely to experience loneliness, than those who reported good health. Those with lower family income were 0.67 more likely to have depression than those with higher income. Those who spent their savings on medical treatment were 1-2x more likely to have anxiety and loneliness than those who did not; those who did not save money for medical expenses were more likely to have depression than those who did. |
| Thayer & Gildner (2020)^45^, United States | Apr 16-30, 2020: First wave with partial restrictions | Pregnant women (*N* = 2099). Age (*M* = 31.3, *SD* 4.4); all female | **Postnatal Depression:** Edinburgh Postnatal Depression Score (EPDS) | **Financial stress:** "I am worried about my financial situation due to the COVID-19 crisis” (5-point agreement scale) | COVID-19-related financial stress was associated with a greater likelihood of experiencing clinically significant depression levels, while controlling demographic characteristics including education and income level (adjusted OR = 2.23, 95% CI = 1.80, 2.77, *p* < .001). |
| Timmins et al. (2022)^54^, United States | Apr 20 and Jul 31, 2020: Initial peak in first wave | Black cisgender sexual minority men and Black transgender women (N = 226). Age (*M* = 25.7, *SD* = 4.0; 88); 88.1% cisgender men, 10.6% transgender women or another gender and assigned male at birth | **Anxiety:** 2 items adapted from General Anxiety Disorder scale (GAD-7) **Depression:** 3 items adapted from the Center for Epidemiologic Studies Depression Scale (CES-D) | **Financial Loss:** "Lost an income source because of the COVID-19 pandemic" (yes, no, no income to lose) **Financial Worries:** "Percentage chance you will lose your job because of the COVID-19 pandemic within 3 months?" (response with percentage) | Income loss and worry that there was a ≥1% chance of losing one's job were associated with loneliness. Income loss and job loss worries were not associated with anxiety and depression. |
| Trógoloet al. (2022)^90^, Argentina | Oct 2020: Peak of first wave | Pre-pandemic workers (*N* = 1049). Age (*M* = 42.15, *SD* = 12.61; 51% male, 49% female | **Burnout:** Maslach Burnout Inventory (MBI-GS) **Depression:** Patient Health Questionnaire (PHQ-9) **Anxiety:** Generalised Anxiety Disorder Scale (GAD-7) **Life satisfaction:** Satisfaction with Life Scale (SWLS) | **Financial hardship:** 1. Income change since COVID-19 (percentage loss) 2. Self-reported financial problems (yes or no) | Workers with financial problems reported greater depression (*F*[1,1045] = 10.53, *p* < 0.001, ηp2 = 0.010) and anxiety (*F*[1,1045] = 6.87, *p* = 0.009, ηp2 = 0.007). Workers with income loss reported lower life satisfaction (F [5,1011] = 2.72, *p* = 0.019, ηp2 = 0.013). |

**Table S6.** *Data Extracted from Longitudinal Studies*

| **Authors, date,**  **country** | **Study period / COVID-19 phase** | **Analytic sample** | **Mental health variable and measure** | **Financial change during COVID-19 variable and measure** | **Main (relevant) findings** |
| --- | --- | --- | --- | --- | --- |
| Badellino et al. (2022),^60^ Argentina | T1: 29^th^ March – 12^th^ April 2020  T2: 23^rd^ May – 12^th^ June 2020,  first wave peaked in October 2020, national lockdown began late-March | General population sample of adults living in Argentina without having previous mental disorder and/ or dyslexia.  **T1**: *N* = 1985, 1505 females, 480 males/other; age: *M* = 36.83 years, *SD* = 14.4  **T2**: *N* = 2839, 2137 females, 702 males/other; age: *M* = 27.95 year, *SD* = 12.23  *N* = 853 respondents participated in both T1 and T2 | **Depressive symptoms:**  Patient Health Questionnaire (PHQ-9) | **Financial worries:**   1. ‘How concerned   have you been about your financial situation in the last month?” A Likert scale from 1 to 10 (0 = no concern and 10 = maximum concern) was used, and the level of concern was ranked as mild (0–4), moderate (5–7) or maximum (8–10)   1. Question about   Concern regarding ‘running out of money to pay expenses, rent and taxes.’ Likert scale from 1 to 4 (1 = not concerned; 2 = little concerned; 3 = concerned; 4 = very much concerned). | Significant increase in the prevalence and average score of depression in Argentine population between late-March and mid-June 2020.  Respondents who expressed concern about running out of money were at a significant risk for depression.  Unemployed participants, who feel more worried about job changes and who were at risk of (or at least concerned about) running out of money to meet their usual expenses, were the most susceptible to depression. |
| Baranov et al. (2022)^20^,  Pakistan | T1: November - December 2019  T2: July 2020, ‘height of the pandemic’ (first peaked in mid-June 2020 in Pakistan), national lockdown ended mid-May and partial restrictions continued | Parents of a school-going child (aged 10-14 years)  (*N* = 883; 725 females, 158 males; age: *M* = 37 years) | **Psychological distress**  (Kessler-10 [K10] questionnaire) | **Economic impact:**  Job loss was used as a proxy for economic impact (respondents were asked if they or their partner lost their job due to the COVID pandemic) | COVID-19-related economic impact was significantly associated with increased psychological distress.  While mental health has not deteriorated overall from baseline during the lockdowns for adults, for those who have suffered economically, it has significantly worsened. |
| Batterham et al.  (2021)^61^, Australia | Seven time points between March – June 2020  (fortnightly surveys), first peak in March/April, national lockdown began in late March, restrictions were eased in early May but local lockdowns and international quarantine continued | General population sample representative of the Australian adult population by age group, gender, and state/territory (quota sampling)  (*N* at T1 = 1296, *N* at T7 = 762 [59%]; 649 females, 647 males; age: *M* = 46.0 years, *SD* = 17.3) | **Depressive symptoms**  Patient Health Questionnaire (PHQ-9)  **Anxiety symptoms**  Generalised Anxiety Disorder (GAD-7) | **Financial distress:**  ‘Over the last 2 weeks, to what extent have you experienced financial distress related to COVID-19?’  (Not at all/A little/ Somewhat/Quite a lot/Considerably/  Extremely) | Mean levels of depression and anxiety symptoms early in the COVID-19 pandemic were higher than estimated by earlier Australian population-based surveys, but most adults did not experience changes in mental health symptoms during the first three months of the  pandemic.  COVID-19-related financial distress was positively associated with higher depression and anxiety at baseline. |
| Bierman et al. (2021)^23^, Canada | T1: March 2020  T2: April 2020  T3: May 2020  T4: June 2020  (monthly surveys), first wave peaked on 30 May 2020, partial national lock-down began in mid-March 2020 | Adults working at time of baseline measures (retained in the sample in subsequent waves if they became unemployed)  (*N* at T1 = 2456, *N* at T4 = 1809 (74%); 1194 females, 1262 males; age: *M* = 41.94 years) | **Psychological distress**  Shortened version of the Kessler-6 [K6] scale | **Economic hardship:**  Measured using 3 questions:   1. ‘How often in   the past month did you have trouble paying the bills?’   1. ‘How   often in the past month did you not have enough money to buy food, clothes or other things your household needed?’   1. How did your   finances work out in the past month? | Positive association between economic hardship prior to the pandemic and periods of economic hardship during the pandemic.  Economic hardship experienced during the COVID-19 pandemic degraded mental health, even when prior mental health and economic hardship was taken into account. Psychological distress predicted economic hardship during the pandemic. |
| Canet-Juric et al. (2020)^55^, Argentina | T1: late-March 2020  T2: April 2020  (12-15 days between surveys), first wave peaked in October 2020, survey launched two days after national lockdown began | General population - 18 years and older, not suffering from physical or psychological illnesses.  (*N* = 6057; 4886 females, 1131 males, 20 ‘other’, 20 ‘prefer not to answer’; age groups:  18-25 = 929  26-40 = 2910  41-60 = 1803  60+ = 415) | **Depressive symptoms**  Beck Depression Inventory (BDI-II)  **State** **anxiety**  State-Trait Anxiety Inventory (STAI)  **Affect**  Positive and Negative Affect Schedule (PANAS) | **Perception of economic impact:** Question regarding variation in economic income due to quarantine (response options: no, few, some, much, very much) | After 2 weeks of quarantine, depression increased, and anxiety and negative and positive affect decreased, all with small effect sizes.  Depressive symptoms increased due to economic hardship.  Lower economic impact was positively associated with more positive affect at both time points.  People who reported no economic impact showed higher positive affect, but larger decrease in positive affect over time. |
| Choi et al. (2023)^21^, USA | T1: 2016  T2: June 2020 (biennial interviews), upswing of first peak, partial restrictions in most states | Nationally representative sample of adults aged 51 years and older and their spouses or cohabiting partners of any age.  (*N* = 1312; 550 females, 762 males; age: *M* = 69.8 years) | **Affect**  International  Positive and Negative Affect Schedule Short-Form (I-PANAS-SF) | **Financial hardship:**  Measured as a count and included missing any regular payment on (a) rent or mortgage, (b) credit cards or other debt, or (c) utilities or insurance; or any indication of difficulty (d) paying medical bills or (e) having enough money to buy food. Observed values ranged from 0 to 5. | During the COVID-19 pandemic, financial hardship was related to increased negative affect and decreased positive affect among, after adjusting for emotional well-being at baseline, and sociodemographic characteristics and health variables at follow-up during the pandemic. |
| Feter et al. (2021)^24^, Southern Brazil (Rio Grande do Sul state) | T1: June – July 2020 but participants were asked to complete MH measure using the period before the COVID-19 pandemic as  reference.  T2: June – July 2020  (retrospective longitudinal design), up to  73.4% of state population were in the second highest level of social distancing restriction | General population sample of adults  *(N* = 2321; 1776 females, 540 males [*n* = 2319]; age groups:  18-30 = 858  31-59 = 1246  60+ = 196 [*n* = 2300]) | **Depressive symptoms**  Hospital Anxiety and Depression Scale (HADS)  **Anxiety**  Hospital Anxiety and Depression Scale (HADS) | **Economic impact:** ‘Did social distancing affect your monthly income?’  In case of an affirmative response, participants asked whether income  decreased or increased during the COVID-19 pandemic. | Prevalence of moderate-to-severe anxiety and depression increased 7.4x and 6.6x, respectively, after the implementation of COVID-19 social distancing restrictions.  A negative economic impact was reported by 45.3% (95% CI: 42.7%, 47.8%) of the respondents.  COVID-19- related income loss was positively associated with higher depression and higher likelihood of more severe anxiety symptoms. |
| Graupensp-erger et al. (2022)^62^,  USA | T1: January 2020  T2: April-May  of 2020  T3: six bimonthly follow-ups from September- October 2020 until July-August 2021; T2: acute early phase, partial restrictions in most states, T3: increase in cases over winter and in July 2021, partial restrictions in most states | Young adults who were aged 18–23 years at screening in 2015-2016.  (Sample for T1 & T2: *N* = 519; 326 females, 193 males/other; age: *M* = 25.4 years, *SD* = 1.84)  (Sample for T3: *N* = 566, 350 females, 216 males/other; age: *M* = 25.8 years; *SD* = 1.83) | **Depressive symptoms**  Patient Health Questionnaire (PHQ-2 or PHQ-8)  **Anxiety**  Generalized Anxiety Disorder Scale (GAD-2 or GAD-7) | **Financial stress:** ‘How concerned are you about the novel coronavirus (COVID-19)….’  Measured on a 5-point Likert-type scale with responses  ranging from ‘not at all’ to ‘extremely’ | Depression symptoms increased between pre- and early-pandemic.  Worse mental health and well-being from pre-pandemic to early-phase-pandemic were positively associated with increased stress in several life domains (e.g., financial stressors and job insecurity stressors.)  Financial stress was uniquely positively associated with symptoms of depression and anxiety across the bimonthly surveys. |
| Hagen et al.  (2023)^56^, Norway | T1: April 2020  T2: December 2020, increasing cases, national restrictions began mid-March 2020 | General population sample of adults living in Norway  (*N* = 6017; 4680 females, 1292 males, 45 other gender; age: *M* = 34.68 years, *SD* = 13.75) | **Depressive symptoms**  Patient Health Questionnaire-9 (PHQ-9)  **Anxiety**  Generalized Anxiety Disorder-7 (GAD-7) | **Negative economic impact:**  Self-reported (yes/no) | Anxiety and depression slightly worsened during the first wave of the pandemic, during national lockdown.  People without pre-existing mental health conditions showed a subclinical increase in symptoms, while people with a pre-exiting mental health condition disorder before the pandemic reported the highest levels of anxiety and depression.  Economic impact was not associated with either depressive or anxiety symptoms. |
| Hertz-Palmor et al. (2021), (study 1)^57^,  International but majority from USA and Israel | T1: 6^th^ April – 5^th^ May 2020  T2: 12^th^ May – 21^st^ June 2020, USA: first wave, partial restrictions in most states; Israel: first wave between March-April, gradual easing of restrictions from early May | General population sample of adults  (*N* = 1318; 1077 females, 241 males; age *M* = 40.79 years,  *SD* = 13.55)  (Reported income loss: *n* = 246) | **Depressive symptoms**  Patient Health Questionnaire-2 (PHQ-2)  **Anxiety**  Generalized Anxiety Disorder-7 (GAD-7) | **Income loss:** Asked whether they had lost their job or whether their pay/ hours were reduced since the beginning of the outbreak. Collapsed into a binary income loss measure (yes/no)  **Financial worry:**  Measured on a 5-point Likert-type scale (from not at all to a great deal) | Income loss due to the COVID-19 pandemic was positively associated with more anxiety and depressive symptoms, but was associated more strongly with depression than anxiety symptoms. The increase in anxiety symptoms was steeper than that of depression symptoms.  There was a positive association between financial worry and depression, whilst controlling for pre-COVID-19 income. This suggests that objective financial situation only partly explains variability in depressive symptoms, and that worries about the financial situation may be a sensitive marker for depressive symptoms during the pandemic. |
| Hertz-Palmor et al. (2021), (study 2)^57^, Israel | T1: 18^th^ – 26^th^ March 2020  T2: 22^nd^ April – 7^th^ May,  first wave between March-April, gradual easing of restrictions from early May | General population sample of adults living in Israel  (*N* = 241; 166 females, 75 males; age: *M* = 37.32 years, *SD* = 12.26; reported income loss: *N* = 102) | **Anxiety**  Patient-Reported Outcomes Measurement Information System (PROMIS) – anxiety items  **Depressive symptoms**  PROMIS depression items | **Income loss:** Measured on a 5-point Likert-type scale (no income loss to extreme income loss)  **Financial worry:** Measured on a 4-point Likert-type scale (from ‘not at all’ to ‘always’) | COVID-19-related income loss and financial worry was positively associated with depression.  COVID-19-related income loss contributed to initial depressive response and its amplification over time, with a 1-month exacerbation in depressive symptoms associated with worsening in income loss.  COVID-19 stress was positively associated with depression but increase in financial worry did not cause an increase in depressive symptoms. |
| Lee et al. (2022)^22^, Singapore | T1: 2017  T2: June 2020 (Contacted if prior measures completed minimum of 6 months prior), two peaks during March and April, strict national restrictions which began to ease in June 2020 | Older Asian adults of Chinese, Malay, or Indian ethnicity, aged 60 years and older (without severe cognitive, hearing, or speech impairment)  (*N* = 496; 272 females, 224 males; age *M* = 73.8 years, *SD* = 7.6) | **Depressive symptoms**  Patient Health Questionnaire (PHQ) | **Financial hardship:**  Economic Hardship Questionnaire (EHQ) with two items removed as these activities could not be undertaken due to lockdown measures  **Changes in lifestyle:**  Due to financial constraints associated with the lockdown (eight items, individual scores used in analyses) | Over a quarter of the sample experienced a decrease in family income, with one‐fifth experiencing financial problems.  Slight reduction in depressive symptoms (after adjusting for covariables) during a 2‐month COVID‐19 lockdown.  Financial hardship was positively associated with depressive symptoms.  Common lifestyle changes made due to financial constraints included: cutting back on charitable contributions (22.3%), changing food shopping or eating habits to save money (11.5%) and reducing household utility use (9.7%). |
| Murray et al. (2023)^19^, Pelotas, city in Southern Brazil | T1: 2015 - 2019  T2: May - September 2020, Pelotas experienced sharp increase in cases, 1 week lockdown, then partial social distancing restrictions | Mothers of 99.9% of all children born in Pelotas in 2015  (*N* = 2083; 2083 females; age groups:  < 20 = 283  20-34 = 1492  ≥ 35 = 308) | **Maternal depressive symptoms**  3 items from Edinburgh Postnatal Depression Scale (EPDS). Rated on a 4-point scale.  (Measured in 2019 and T2)  **Maternal anxiety**  Generalized Anxiety Disorder (GAD-7)  (Measured in 2016 and T2) | **Income loss:** Asked whether family income in the last month ‘got a lot worse,’ ‘got a little worse,’ ‘stayed the same,’ or ‘got better’. Coded as ‘yes’ (‘got a lot worse’) or ‘no’ for analyses.)  **Emergency welfare receipt:**  Only made to families with financial difficulties. Coded as ‘yes’ or ‘no’ to question asking whether this had been received by someone in the household. | During the COVID-19 pandemic, maternal depressive symptoms increased substantially, while there were small decreases in maternal anxiety.  Both COVID-19-related income loss and emergency welfare receipt during the pandemic were positively associated with maternal depression and maternal anxiety, after adjusting for both baseline levels of these variables and pre-pandemic family income. |
| Shuster et al. (2021)^58^, USA | Weekly surveys for 10 weeks between 2^nd^ April – 4^th^ June 2020, first wave of COVID-19, partial restrictions in most states | General population sample of USA residents aged between 18–64  (*N* = 743; 359 females/other, 384 males; age: *M* = 37.1 years) | **Depressive symptoms**  Zung Self-Rating Depression scale  **Anxiety**  State Anxiety Inventory | **Economic impact:** ‘Rate the impact that COVID-19 has had on your economic situation’ (rated from very negative impact, −50 to very positive impact, +50, scaled to be between −0.5 and 0.5 for analysis) | Depression and anxiety initially peaked but then declined over 10 weeks during the first wave of COVID-19.  COVID-19-related economic impact was positively associated with depressive and anxiety symptoms. |
| Simonse et al. (2022)^1^, Netherlands | T0: April – November 2018  T1: December 2019 – March 2020  T2: December  2020 – March 2021, T2: increasing cases of new variant, national lockdown in place | Population representative sample of residents  (T0: *N* = 1114; 613 females, 501 males/  other; age: *M* = 53.0 years, *SD* = 17.8)  (T1: n = 838; 451 females, 387 males/  other; age: *M* = 54.5 years, *SD* = 16.9)  (T2: *N* = 736; 390 females, 346 males/ other; age: 55.6 years, *SD* = 16.6) | **Mental health**  Mental Health Index (MHI-5) | **Financial stress:**  Psychological Inventory of  Financial Scarcity (PIFS); responses range from 1 (totally disagree) to 7 (totally agree). | Mean levels of mental health did not change in the first six months of the pandemic compared to pre-pandemic. This, however, masked underlying heterogeneity as for four out of five respondents, mental health either increased or decreased. Increase in financial stress predicted worsened mental health, and vice versa. Financial stress mediated the relation between savings and debts together, and mental health. |
| Strizzi et al. (2023)^59^, Denmark | T1: October – November 2020  T2: March - May 2021, increasing cases, national restrictions began 12^th^ March 2020 | Sample drawn from nationally representative sample of 5,000 Danish residents aged >18 (*N* = 1,302)  (**T1**: *N* = 914; 493 females, 421 males; age: *M* = 51.29 years, *SD* = 16.67)  (**T2**: *N* = 304; 154 females, 150; age: *M* = 56.38 years, *SD* = 15.82)  (**Both** **T1 and T2**: *N* = 84; 47 females, 37 males; age: 52.22 years, *SD* = 14.05) | **Depressive symptoms**  Patient Health Questionnaire (PHQ-9)  **Anxiety**  Generalized Anxiety Disorder (GAD-7) | **Loss of income:**  ‘Since the COVID-pandemic, have you personally experienced a loss of income?’ (response options: 1 = *yes, a total loss of income*, 2 = *yes, a partial loss of income*, 3 = *no loss of income*, 4 = *I had no personal income before COVID-19*). Response options dichotomized for analyses: 0 = no income loss (3–4) and 1 = income loss (1–2). | Mean levels of anxiety and depressive symptoms did not change during the first year of the pandemic and did not differ from those of matched controls assessed before the pandemic. After adjusting sociodemographic variables (e.g., age, gender) COVID-19-related income loss was positively associated with depression and anxiety symptoms. |
| Weber et al. (2023)^63^, Germany | T1: May 2020  T2: September 2020  T3: December 2020  T4: March 2021  T5: March 2022,  T3 = height of COVID-19-related death rate, first national lockdown on 23^rd^ March – May 2020, second lockdown November 2020 – May 2021 | General population sample of adults living in Germany  (**T1**: *N* = 636; 535 females, 85 males, 15 diverse gender, 1 missing; age: *M* = 39.5 years, *SD* = 16.11)  (**T5**: *N* = 216; 176 females, 31 males, 9 diverse gender, 1 missing; age: *M* = 40.48 years, *SD* = 17.03) | **Depressive symptoms**  Patient Health Questionnaire (PHQ-9)  **Anxiety**  Generalized Anxiety Disorder Scale (GAD-7) | **Financial worry:**  ‘During the past 4 weeks, have you worried about your financial situation?’  (responses include ‘not at all’, ‘not more than usual’, ‘more than usual’, much more than usual’) | Depression and anxiety declined following the lifting of lockdown measures, peaked during the two national lockdown phases in Germany, and dropped during the easing phases. Initial financial worry due to COVID-19 was positively associated with anxiety and depression at each time point.  Initial financial worry due to COVID-19 was positively associated with greater symptom decreases in anxiety and depression across the pandemic. |

Abbreviations: T = time point (e.g., T1 = time point 1)

**Figure S1**

*Funnel Plot for Anxiety Meta-Analysis*

**Figure S2**

*Funnel Plot for Depression Meta-Analysis*
